# Optical Properties and Molecular Composition of Fine Organic Aerosols in Nanjing, China: A Comparison of 2019 and 2023

**DOI:** 10.3390/toxics13060443

**Published:** 2025-05-27

**Authors:** Binhuang Zhou, Yu Huang, Liangyu Feng, Zihao Zhang, Haiwei Li, Yun Wu, Jianhuai Ye, Xinlei Ge

**Affiliations:** 1 Jiangsu Key Laboratory of Atmospheric Environment Monitoring and Pollution Control, Collaborative Innovation Center of Atmospheric Environment and Equipment Technology, School of Environmental Science and Engineering, Nanjing University of Information Science and Technology, Nanjing 210044, China; 2 School of Environmental Science and Engineering, Southern University of Science and Technology, Shenzhen 518055, China; 3 School of Environment and Energy Engineering, Anhui Jianzhu University, Hefei 230601, China

**Keywords:** molecular composition, light absorption, fluorescent properties

## Abstract

Optical properties and chemical composition of atmospheric fine particles (PM_2.5_) are critical to their environmental and health effects. In this study, we analyzed the organic aerosols (OA) in PM_2.5_ samples in Nanjing, China, collected during the summer and winter of 2019 and 2023. Results show a decline in both concentrations and light-absorbing abilities of methanol—soluble organic carbon (MSOC) and water-soluble OC (WSOC) in OA from 2019 to 2023. Due to increased combustion activities, MSOC and WSOC concentrations, and their corresponding mass absorption efficiencies were all higher in winter than in summer. Furthermore, fluorescence indices suggest that OA in Nanjing was influenced by a mix of microbial/biogenic sources. Fluorescent properties of both WSOC and MSOC were dominated by humic-like components but the remaining contribution from protein-like components was more significant in MSOC. The molecular composition of OA did not show a remarkable difference between 2019 and 2023. Overall, CHON compounds were the most abundant species, followed by CHO and CHN compounds, and aliphatic compounds dominated all molecular types except for CHN (in positive mode) and CHON, CHOS (in negative mode). Regarding the OA sources, the numbers of molecules from fossil fuel combustion and biomass burning (BB) were a bit more in 2023 than in 2019, and signal intensities of BB-related molecules were also higher in winter than in summer; the presence of organosulfates indicate the contribution of aqueous-phase oxidation to OA, especially during high relative humidity conditions. At last, correlations between OA molecules and light absorption efficiencies indicate that the key light-absorbing species in winter and summer were likely quite different despite similar chemical compositions, and in summer, CH and CHN compounds were important to light absorption, whereas CHNS compounds became more important in winter.

## 1. Introduction

Organic aerosol (OA) is a major and highly complicated component of atmospheric aerosols, accounting for 20% to 90% of their total mass [1], and playing a crucial role in air quality, climate change and human health. OA includes species emitted from multiple primary sources, including biomass burning, biofuel combustion [2,3], fossil fuel combustion (such as traffic activities) [4,5,6,7], and species from various secondary chemical reactions [2,8,9,10,11,12]. Many OA species can absorb ultraviolet-visible (UV-Vis) lights, which are collectively termed “brown carbon” (BrC), and are an important driver of aerosol’s radiative effect [13,14,15,16]; for example, it can occupy 30–50% of the total OA light absorption of in 300–400 nm wavelength range [5,17]; it also affects the cloud condensation nuclei (CCN), therefore cloud formation and climate indirectly [18,19]. BrC mainly includes some highly conjugated organic compounds, such as polycyclic aromatic hydrocarbons (PAHs), humic-like substances (HULIS), etc. [20,21,22]; a recent study shows that nitrogen-containing organics dominate the light absorption of OA [23]. The light absorption properties of OA are typically measured by the ultraviolet-visible (UV-Vis) spectrophotometry [16,24]. The excitation-emission matrix (EEM) fluorescence spectroscopy is on the other hand used to determine the fluorescence properties of OA, and further parallel factor analysis (PARAFAC) can infer compositions of its key fluorophores [25,26,27]. The molecular structure of OA has a significant impact on its optical characteristics [28] yet due to the complex composition and diverse sources of OA, our understanding remains poor.

Offline analysis of OA requires proper pre-treatment of the samples at first. Various solvents can be used to extract the OA, and organic solvents (such as methanol) typically offer significantly higher extraction efficiencies than water [29]. Some studies show that water-insoluble organic carbon (WISOC) can contribute substantially to OA’s light absorption, and have a mass absorption efficiency potentially larger than that of water-soluble OA [22,30,31,32,33,34]. Three-dimensional fluorescence spectra of OA extracted using different solvents, and following identified fluorophores also exhibit differences [27,33,35]. Therefore, OA extraction by organic solvents such as methanol can achieve a relatively comprehensive analysis of the composition and optical properties of OA. Subsequently, molecular characterization of OA is often fulfilled by using non-targeted high-resolution mass spectrometry coupled with liquid chromatography (LC) or gas chromatography (GC) [36,37]. This approach, together with key parameters like double bond equivalents (DBE) and elemental ratios of the identified molecules, offers valuable insights into the understanding of OA chemical properties. Relatively, some polar compounds and compounds with medium weight are easier to identify due to high ionization efficiency. For instance, Wang et al. (2018) observed notable differences in the chemical composition of OA between Beijing and Mainz, with Beijing showing a higher number of identified compounds particularly combustion-related aromatic hydrocarbons [38]. Wang et al. (2021) found that the molecular compositions of OA in Taiyuan, China, exhibited distinct seasonal variations [39], with more organosulfates in summer, resulting in a more oxidized and saturated OA. Mao et al. (2022) investigated the molecular composition of OA in coal combustion-polluted areas and identified nitrogen-containing organics as an important component [37], and those nitro-PAHs containing one nitrogen as markers of coal combustion emissions.

Nanjing is a densely populated megacity in the Yangtze River Delta (YRD) region in China, therefore fine particulate matter (PM_2.5_) pollution is a critical environmental issue. Although there are some results available that analyzed the chemical composition, optical properties, health risks, and sources of fine particles in Nanjing including a number of earlier studies from our group [40,41,42], understanding of the optical and chemical properties of ambient OA in Nanjing is still incomplete, especially the molecular level chemical composition. To address this, this work collected and measured the optical properties and molecular composition of PM_2.5_ samples during the summer and winter of 2019 and 2023, respectively. The comparison of 2019 and 2023 also offers insights into the changes in OA properties before and after the three-year COVID-19 influences in China. Elucidation of chemical characteristics of OA serves also as a basis for further toxicological studies.

## 2. Experimental Methods

### 2.1. Sample Collection and Pre-Treatment

PM_2.5_ samples were collected from the rooftop of the library building (~21 m above the ground) in Nanjing University of Information Science and Technology (32.20° N, 118.71° E), Nanjing, China. The site locates in suburban Nanjing, near major traffic roads, and is influenced by a mix of industry, traffic, residential and agricultural emissions. A high-flow sampler (KB-1000, Jinstar, Qingdao, China) was used to collect PM_2.5_ samples at a flow rate of 1.05 m^3^/min. The samples were collected on pre-baked quartz filters (at 450 °C for 4 h) to remove potential contaminants. Blank samples were processed in the same way as background. The sampler uses a cutter to separate PM_2.5_ by removing particles larger than 2.5 μm based on the principle of inertia. Afterwards, the air carrying PM_2.5_ continues to move forward and reaches the quartz filter with a specific pore size, on which PM_2.5_ are intercepted. Each PM_2.5_ sample was collected for 22 h from 12:00 PM to 10:00 AM of the following day. Samples were not collected during precipitation. In total, 193 samples were obtained: 1 June–31 August 2019 (73 samples), 3 December 2019–20 January 2020 (38 samples), 2 June–26 August 2023 (52 samples), and 5 December 2023–28 February 2024 (30 samples). After collection, the PM_2.5_ mass concentrations on filters were immediately determined using a digital balance (OHAUS DV215CD, Parsippany, NJ, USA; precision 0.01 mg). The filters were then wrapped in clean aluminum foils and stored in the refrigerator (−20 °C) before analysis. Throughout the sampling period, meteorological parameters, including temperature, relative humidity, wind speed, and wind direction, were recorded by a weather station located ~50 m from the sampling site.

Round pieces of the filters, each of 25.13 cm^2^ and 9.08 cm^2^, were punched from the filters, and extracted using 40 mL of ultrapure water (18.2 MΩ·cm, TOC < 5 ppb) and 20 mL of methanol (MERCK, Rahway, NJ, USA), respectively. Ultrasonic extraction was performed in an ice bath for 30 min, followed by filtration through a 0.45 μm filter, to remove and minimize possible influences of the insoluble substances For methanol-extracted samples, the filters were air-dried overnight in a fume hood, wrapped in clean aluminum foils and stored in a desiccator until further analysis of organic carbon (OC) and elemental carbon (EC). Blank samples were processed in the same manner to ensure consistency.

### 2.2. Chemical Analysis

The concentrations of water-soluble organic carbon (WSOC) in samples were determined using a total organic carbon (TOC) analyzer (TOC-L, Shimadzu, Kyoto, Japan). OC and EC contents were determined by a thermal-optical OC/EC analyzer (Sunset Laboratory, Portland, OR, USA), using the round piece of 2.01 cm^2^ (17 mm diameter) for each filter. OC and EC contents of the filters after methanol extraction were also measured. Methanol-soluble organic carbon (MSOC) was then calculated as the difference of OC concentration before and after extraction. More details are provided in our previous work [43,44]. Measured OC, EC, WSOC and MSOC concentrations are used for calculation of optical properties (Section 2.3).

The molecular composition of OA was measured by a high-performance liquid chromatography (HPLC) coupled with a quadrupole time-of-flight mass spectrometer (Q-ToF-MS). We selected six samples (three with high and three with low PM_2.5_ concentrations) in each period (in total 24 samples) for analysis. In details, five round pieces (3.14 cm^2^) of each filter were subjected to extraction with 5 mL methanol (Optima LC/MS grade, Fisher Chemical, Waltham, MA, USA) ultrasonically at room temperature for 30 min. The extraction was repeated three times. Afterwards, the samples were filtered through a 0.22 μm PTFE filter and blew dried by nitrogen gas. The residue was re-dissolved in 300 μL methanol and stored in a 1.5 mL amber vial. Compound separation was performed using a Luna Omega C18 (PHENOMENEX, Torrance, CA, USA) 1.6 μm C18 column (100 mm × 2.1 mm × 1.6 µm) at 40 °C (a sample chromatogram is provide in Figure S1a). The electrospray ionization (ESI) was conducted in both positive and negative ion modes. The mass-to-charge (*m*/*z*) scanning range was 50–1100 amu, with an interval of 1 s. The ionization parameters were: drying gas temperature at 320 °C, nitrogen gas flow rate at 8 L/min, sheath gas flow rate at 11 L/min, sheath gas temperature at 350 °C, and capillary voltages at 4000 V (positive mode) and 3500 V (negative mode).

In positive mode, the mobile phase was consisted of solvent A (ultrapure water containing 0.1% *v*/*v* formic acid) and solvent B (acetonitrile containing 0.1% *v*/*v* formic acid). The eluent was programmed as: 0–1 min: 5% B; 1–3 min: linearly increase to 25% B; 3–20 min: linearly increase to 75% B; 20–50 min: linearly increase to 100% B; 50–53 min: maintain at 100% B; 53–55 min: decrease to 5% B. In negative mode, the mobile phase was consisted of solvent A (ultrapure water containing 0.05% *v*/*v* ammonia) and solvent B (acetonitrile containing 0.1% *v*/*v* formic acid). The eluent was set as 50% solvent A and 50% solvent B in the first 2 min, after that the sample was directly injected into the mass spectrometer.

The data were processed by the MS-DIAL software (version 4.92) [45], which includes peak extraction, peak alignment, and deconvolution. The ion fragments analyzed included [M−H^−^] [M+H^+^], [M+NH_4_^+^], and [M+Na^+^]. All deconvoluted spectra were exported to MS-FINDER (version 3.52) to assign the molecular formulas (the sample mass spectra for both positive and negative modes are provide in Figure S1b,c respectively). Several constraints were then applied to exclude unrealistic formulas: (1) Atomic numbers: C: 1–50, H: 1–100, O: 0–40, N: 0–5, S: 0–2; (2) Elemental ratios: H/C: 0.3–3.0, O/C: 0–1.2, N/C: 0–1.3, S/C: 0–0.5; (3) Double bond equivalence (DBE): 0–25. DBE is used to indicate the unsaturation degree [46] with the following formula:

(1) $$DBE=\frac{\left(2\times C-H+N+2\right)}{2}$$

where C, H, and N represent the numbers of carbon, hydrogen, and nitrogen elements in the molecular formula. Additionally, the aromaticity equivalent (Xc) is widely used to distinguish aliphatic compounds from aromatic and polycyclic aromatic compounds [47], as follows:

(2) $$Xc=\frac{3\times \left(DBE-\left(p\times O+q\times S\right)\right)-2}{DBE-\left(p\times O+q\times S\right)}$$

where *p* and *q* represent the fractions of oxygen and sulfur atoms involved in the π bond structure, respectively. In this study, for substances in ESI− mode, *p* = *q* = 0.5, and for those in ESI+ mode, *p* = *q* = 1 [48,49]. Compounds with Xc < 2.5 are considered to be aliphatic, those with Xc ≥ 2.5 are aromatic, and compounds with Xc ≥ 2.7 are classified as polycyclic aromatic compounds [47]. The carbon oxidation state (OSc) [50], defined as 2 × O/C-H/C, can be used to describe the oxidation degree of molecule. Equations (1) and (2) were applied for each individual identified peak in the chromatogram of each sample.

Moreover, based on the relative abundances of identified molecules, the average O/C, H/C, and DBE values of each sample were calculated using the following formula [51]:

(3) $$\frac{O}{C}=\frac{\sum \left({lin}_{i}\times {\frac{O}{C}}_{i}\right)}{\sum {lin}_{i}}$$

(4) $$\frac{H}{C}=\frac{\left(\sum {lin}_{i}\times {\frac{H}{C}}_{i}\right)}{\sum {lin}_{i}}$$

(5) $$DBE=\frac{\left(\sum {lin}_{i}\times {DBE}_{i}\right)}{\sum {lin}_{i}}$$

Here, *lin* represents the relative abundance of molecule *i*, and O/C*_i_*, H/C*_i_*, DBE*_i_* are the corresponding values of molecule *i*.

### 2.3. Optical Analysis

The light absorption of both water and methanol extracts were measured in 200–800 nm wavelength using a UV-Vis spectrophotometer (UV-3600, Shimadzu, Japan). The light absorption coefficient (Abs) at a specific wavelength (λ) was calculated using the following Equation (6):

(6) $${Abs}_{\lambda }=\frac{\left(A_{\lambda }-A_{700}\right)V_{1}}{V_{a}\times l}\times \ln\left(10\right)$$

Here, A_700_ is used to account for baseline drift. V_1_ is volume of the solvent, V_a_ is volume of the corresponding sampled air, and l is the path length of the quartz cuvette (1 cm here).

The wavelength dependence of the light absorption can be represented by the Ångström Absorption Exponent (AAE) [18], as shown in the following Equation (7):

(7) $${Abs}_{\lambda }=K\times \lambda^{-AAE}$$

where *K* is a dimensionless constant related to light absorption, and the range of λ here is 300–450 nm.

The mass absorption efficiency (MAE, m^2^ g^−1^) at 365 nm (values at this wavelength can effectively eliminate interferences of inorganic salts) [19,27,52,53] can be calculated below (4):

(8) $$MAE=\frac{{Abs}_{365}}{WSOC\left(MSOC\right)}$$

The imaginary part of the particle refractive index, k can be used to represent the light absorption rate of atmospheric aerosols [19], and is expressed as follows (9):

(9) $$k=\frac{{MAE}_{\lambda }\times \lambda \times \rho }{4\pi }$$

where *ρ* (g cm^−3^) is the particle density, which is set to 1.5 in this study [9].

For the fluorescent properties, the EEM spectra were determined by a fluorescence spectrophotometer (Cary Eclipse, Agilent, Santa Clara, CA, USA). The excitation (Ex) wavelength was 235–500 nm with an interval of 5 nm, and the emission (Em) wavelength was 300–600 nm with an interval of 2 nm. The scanning speed was 1200 nm min^−1^, with a slit width of 5 nm. Systematic biases, Raman and Rayleigh scattering, as well as blanks from water and methanol, were corrected. Then, the fluorescence data were processed using the PARAFAC model and drEEM toolbox (version 0.6.5, MATLAB R2018a) [54]. The model was run with non-negative constraints, and a half-split analysis.

Additionally, a few indices were calculated to investigate the fluorescence properties of OA. The humification Index (HIX) is the ratio of integrated emission signal of 436–480 nm to that of 300–344 nm at an excitation wavelength of 255 nm. The fluorescence index (FI) is the ratio of emission at 470 nm to 520 nm upon excitation at 370 nm. The biological index (BIX) is the ratio of emission at 380 nm to 430 nm upon excitation at 310 nm [55,56].

## 3. Results and Discussion

### 3.1. Light Absorption Properties

Table 1 lists the average concentrations (OC, EC, WSOC and MSOC) and light absorption parameters of OA during different periods. First of all, we need to conduct the significance test on the different pairs of data so that comparison and relevant interpretation are reliable. Before that, the tests of normality distribution were conducted and results are shown in Table S1, which shows that some data (for example, EC concentrations in all periods) do not follow the hypothesis of normality distribution, therefore the Wilcoxon test rather than the two-sample *t*-test is more suitable to our dataset. The corresponding results are presented in Table S2. The *p* values of four pairs of data (summer 2019 vs. winter 2019, summer 2019 vs. summer 2023, summer 2023 vs. winter 2023, winter 2019 vs. winter 2023) are less than 0.05 (on 95% confidence interval) (many items are actually close to zero). These statistical tests support our discussion and interpretation on the measurement data are reliable.

**Figure 1:**
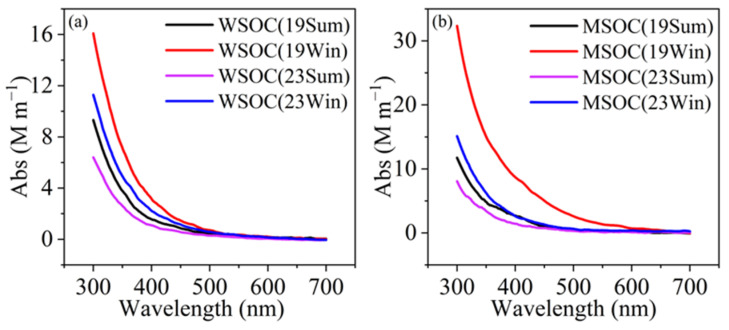
The average light absorption spectra of (**a**) WSOC and (**b**) MSOC during four periods.

As expected, winter levels were higher and that of 2019 winter was the highest. The mean methanol extraction efficiency (MSOC/OC) (0.80~0.90), was significantly higher than that of water (0.56~0.63), affirming that MSOC can better represent OA. Correspondingly, across the measured wavelength range (300–700 nm), the light absorption of MSOC was also noticeably higher than WSOC (Figure 1), consistent with prior findings in Seoul, South Korea [8], Beijing [29], Xi’an [22], and Nanjing [27], etc. For the values at 365 nm, Abs_MSOC-365_ were generally higher than Abs_WSOC-365_ during all four periods; and again, those during winter were higher than those in summer, similar to previous observFations [8,27,53], and those of 2023 became smaller than those of 2019. Figure S2 further presents scatter plots among Abs_365-WSOC_ and MSOC, where the tight correlations suggest a large degree of overlap between MSOC and WSOC.

**Figure 2:**
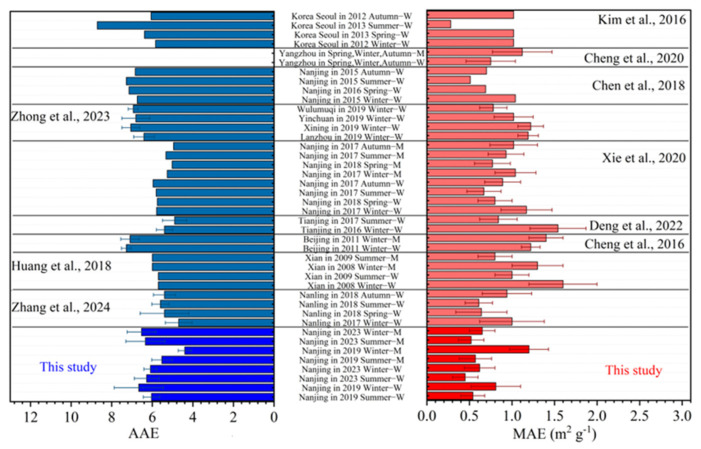
The values of Ångström absorption exponent (AAE) and mass absorption efficiency (MAE) in this study and other key selected studies (Zhong et al., 2023 [58], Huang et al., 2018 [22], Zhang et al., 2024 [57], Kim et al., 2016 [8], Chen et al., 2020 [53], Chen et al., 2018 [52], Xie et al., 2020 [27], Deng et al., 2022 [63], Cheng et al., 2016 [29]) (the name of campaign with a suffix of “-M” means that the data is for MSOC, while that with a “-W” means it is for WSOC) (blue bars represent AAE values while red bars represent MAE values).

As shown in Table 1 and Figure 2, the MAE of methanol extracts were higher than those of water extracts as well. This result aligns with previous studies [8,21,27], suggesting that the water insoluble OC (WIOC) exhibits a stronger light absorption capacity and can be efficiently extracted by methanol. The MAE values observed in this study are comparable to those from a South China site in Nanling (summer: MAE_WSOC-365_ = 0.61 ± 0.16 m^2^ g^−1^, winter: MAE _WSOC-365_ = 1.0 ± 0.38 m^2^ g^−1^) [57], and from our previous results in Nanjing (summer: MAE _WSOC-365_/MAE _MSOC-365_ = 0.67 ± 0.20/0.93 ± 0.21, winter: MAE_WSOC-365_/MAE_MSOC-365_ = 1.17 ± 0.30/1.04 ± 0.24) [27], but are lower than those in northern China, such as Beijing (winter: MAE_WSOC-365_ = 1.22 ± 0.11 m^2^ g^−1^, MAE_MSOC-365_ = 1.40 ± 0.20 m^2^ g^−1^) [29], Lanzhou (winter: MAE_WSOC-365_ = 1.19 ± 0.12 m^2^ g^−1^), Xining (winter: MAE_WSOC-365_ = 1.22 ± 0.18 m^2^ g^−1^), Yinchuan (winter: MAE_WSOC-365_ = 1.02 ± 0.23 m^2^ g^−1^), and Xi’an (summer: MAE_WSOC-365_/MAE_MOC-365_ = 0.98 ± 0.21/1.65 ± 0.36, winter: MAE_WSOC-365_/MAE_MSOC-365_ = 0.78 ± 0.23/1.33 ± 0.34) [58]. This result likely suggests that light absorption ability of OA in South China is generally weaker than it in North China.

Unlike black carbon (BC), BrC has strong wavelength dependence and different species have significantly different average AAE values (Figure 2). As shown in Figure 2, the AAE values for WSOC and MSOC observed in this study (marked in the bottom part) are comparable to those reported in previous studies in Nanjing (4.95–5.97) [27], Seoul (4.09–8.71) [8], etc. However, the values are lower than those reported in winter Beijing (7.1 ± 0.45 for MSOC) [29] and United States (7.63–8.71) [59]. Some studies report AAE values of wood burning particles to be 6.9–11.6 [16], and those from laboratory simulated secondary reactions are 5.2–8.8 [60]. Since the AAE values observed here are generally lower than 7, likely indicating a dominance of secondary sources to both WSOC and MSOC. MSOC appeared to have smaller AAE values than those of WSOC (Table 1), indicating that the WIOC has much weaker wavelength dependence than the WSOC [61]. MSOC AAE values in summer were larger than those in winter for both 2019 and 2023, while the differences of WSOC AAE in different periods were insignificant. In addition, The MSOC AAE values were higher in 2023 than those in 2019, both in summer and winter, suggest a substantial change in sources and compositions of MSOC rather than WSOC before and after the three COVID-19 years.

The imaginary refractive index (*k*) is another important parameter to assess the direct radiative forcing of aerosols in climate models [18,62]. At 365 nm, average k_MSOC-365_ in Nanjing during the summer (winter) of 2019 were 0.0247 (0.0525), while in 2023, it ranged from 0.0226 (0.0284) (Table 1). These variations follow the same trend as the MAE_365_, consistent with previous studies [63,64]. The k_365_ value of this study is slightly lower than that in northern Chinese cities such as Jinan (0.035) [65], Lanzhou, Xining, Yinchuan, and Urumqi (0.023–0.034) [58], and in Kanpur, India (0.042) [66], but higher than those in Himalayan cryosphere (0.009–0.026) [67]. In addition, previous studies have shown that photochemical aging may lower the k_365_ values [19,68]. Both MAE_365_ and k_365_ exhibited significant seasonal changes, reflecting the differences regarding primary sources and secondary processes (such as photo-bleaching) of OA across seasons. The higher MAE_365_ and k_365_ values in winter were likely due to increased anthropogenic activities such as biomass and coal burning. Moreover, the overall decreases in MAE_365_ and k_365_ in summer and winter of 2023 compared to 2019 demonstrates a decline of the atmospheric light absorption capacity of OA.

### 3.2. Fluorescent Properties

#### 3.2.1. Fluorescent Indicators

FI is often used as an indicator of organic sources in water and soil environments [69,70], and now it has been applied in aerosol chemistry [63,71,72]. FI < 1.4 indicates the terrestrial origin of OA, while FI > 1.9 indicates a dominance of microorganisms, and in general FI is inversely proportional to the aromaticity of OA. BIX in general reflects the relative contribution of recently emitted organics (such as those from microbial activities), therefore a high BIX indicates a high contribution of fresh organics [73]. HIX can reflect the degree of humification [55,56], and a higher HIX typically refers to a higher OA aromaticity [54,74].

**Figure 3:**
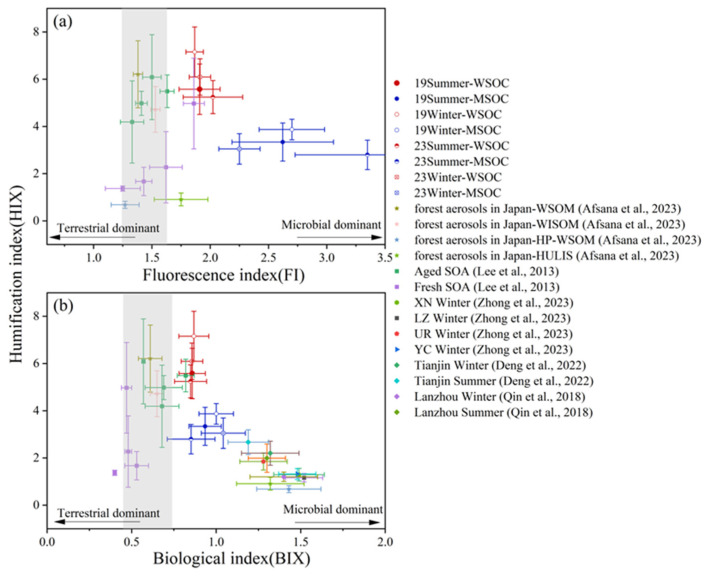
Distribution of Fluorescence Indices in This Study Compared with Other Studies (Afsana et al., 2023 [35]; Lee et al., 2013 [70]; Zhong et al., 2023 [58]; Deng et al., 2022 [63]; Qin et al., 2018 [72]): (**a**) Fluorescence Index (FI) vs. Humification Index (HIX), (**b**) Biological Index (BIX) vs. Humification Index (HIX). The shaded areas represent BIX values from 0.6 to 1 [69] and FI values from 1.6 to 1.9 [56].

As shown in Figure 3 and Table 2, the mean HIX values for WSOC were high (5.58 ± 1.07 in summer, 7.16 ± 1.06 in winter in 2019; 5.24 ± 0.70 in summer, 6.09 ± 0.77 in winter in 2023), much higher than those in Lanzhou (2.0 in summer and 1.2 in winter) [72] and Tianjin (3.12 in summer and 2.47 in winter) [63], but in agreement with previous results in Nanjing, China (6.97 in summer and 7.20 in winter) [27]. The variations of HIX_MSOC_ was similar to that of WSOC in the four periods, and the average HIX_MSOC_ values were much lower than HIX_WSOC_, suggesting that water-extracted OA has a much higher aromaticity than its methanol-extracted companion. For both WSOC and MSOC, the mean FI values were higher in summer than in winter, but different from HIX, mean FI_MSOC_ values (2.25–3.35) were all higher than the FI_WSOC_ values (1.87–2.02) in all four periods. This result indicates the WIOC dissolved in methanol rather than water might be associated with biogenic sources. The FI_WSOC_ values in this study are similar to our earlier results in Nanjing (1.82 in summer and 1.91 in winter) [27], and those of MSOC are slightly higher (1.54 in summer and 2.28 in winter for MSOC). For BIX, the differences for both WSOC and MSOC values across all sampling periods were small (0.85–0.87 for WSOC, and 0.85–1.04 for MSOC). As shown further in Figure 3, most BIX values distributed within the range of 0.6 to 1 [69], while the FI values distributed in the range of >1.6 [56], indicating that the OA in summer and winter of both 2019 and 2023 were influenced by microbial/biogenic source.

In addition, the HIX values in 2023 for both WSOC and MSOC appeared to drop significantly from those in 2019, demonstrating a big decrease of OA’s aromaticity in Nanjing. Except for MSOC in winter, the FI values were larger in 2023 than in 2019, which are consistent with that FI is inversely related to aromaticity. The 2023 to 2019 difference of BIX was, not significant.

#### 3.2.2. Key Fluorescent Components

**Figure 4:**
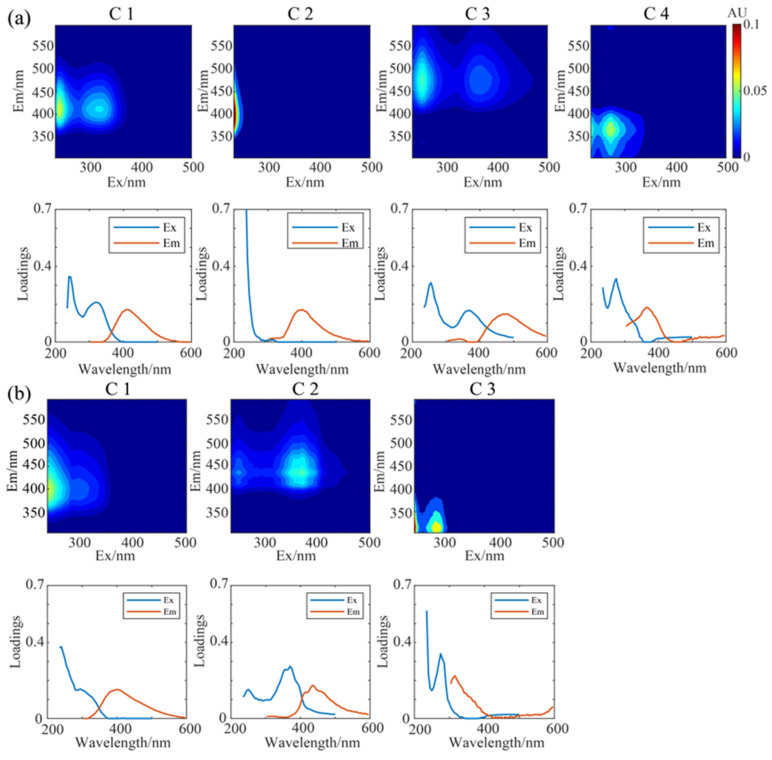
The florescent components resolved by the PARAFAC analyses and its excitation and emission wavelengths: (**a**) WSOC (C1–C4) and (**b**) MSOC (C1–C3).

The key fluorescent components resolved by the PARAFAC analysis on the 3D-EEM spectra are shown in Figure 4. Four components were identified for WSOC. C1 exhibited two characteristic peaks at Ex/Em = 240/410 nm and Ex/Em = 320/410 nm. Generally, a bi-modal distribution in the fluorescence spectra indicates the presence of HULIS [75,76,77], and the second peak at relatively long excitation wavelengths refers to condensed aromatic substances, conjugated structures, or non-linear cyclic systems. C3 displayed a similar peak pattern therefore was also linked with HULIS, but with higher excitation and emission wavelengths (Ex/Em = 255/480 nm and Ex/Em = 370/480 nm), suggesting a higher aromaticity. C3 likely contains a richer conjugated unsaturated structure, while C1 is more likely associated with photodegradative decomposition of macromolecules [33]. In contrast to C1 and C3, C2 exhibited a small characteristic peak at Ex/Em ≤ 235/400 nm, also classified as a HULIS component, but likely contains more oxygenated structures and may have a lower degree of oxidation [78]. C4 was identified as a protein-like component, exhibiting characteristic peaks at Ex/Em ≤ 235/364 nm and Ex/Em = 275/364 nm (shorter wavelengths) [75]. Previous studies have shown that tryptophan-like proteins have excitation wavelengths in the range of 220–290 nm and emission wavelengths in the range of 320–380 nm [79]. Therefore, C4 of WSOC here likely associates with tryptophan-like fluorescent chromophores [54] and may be relevant with anthropogenic activities [33,69,80]. Figure S3a presents the average contributions of these four components in different periods, C2 was the most abundant component especially in winter, followed by C1 whose contribution increased significantly in summer, C3 and C4 were minor components.

For MSOC, only three key fluorophores were separated (Figure 4b). C1 and C2 were classified as HULIS while C3 was a protein-like component. C1 exhibited characteristic peaks at Ex/Em = 235/402 nm and Ex/Em = 275/402 nm, similar to C1 of WSOC but with slightly lower excitation and emission wavelengths. C2 had prominent peaks at Ex/Em = 250/436 nm and Ex/Em = 370/436 nm, similar to C3 of WSOC but with a larger Stokes shift. The right-side peak has stronger intensities, indicating possible presence of more conjugated molecules, with larger molecular weights and conjugated bonds, and condensed aromaticity [77]. We showed earlier that MSOC HIX was lower than WSOC HIX in both summer and winter, consistent with the lower emission wavelength of MSOC than WSOC here, reaffirming the higher aromaticity of WSOC than MSOC. C3 had characteristic peaks at Ex/Em ≤ 235/316 nm and Ex/Em = 275/316 nm. Its maximum emission wavelength was lower than the protein-like component C4 of WSOC, and was not within the range of aro-matic proteins (330–380 nm), instead lay between those of tryptophan and tyrosine. We postulate that C3 may include not only proteins (such as tyrosine and tryptophan), but also phenolic compounds or PAHs from biomass and/or fossil fuel combustion [53,80,81,82,83]. For their contributions in different periods (Figure S3b), C1 became the most abundant component and was slightly more abundant in summer than in winter; the protein-like C3, very differently from that in WSOC, was also rich especially in winter (up to 40.3% in 2019 winter); C2 on the contrary, was the least important component. Overall both MSOC and WSOC fluorescence were dominated by HULIS, but the large difference regarding fluorescent properties between the two, despite their possible large overlap of chemical species, suggests a mismatch of chemical species and fluorophores.

### 3.3. Molecular Composition

#### 3.3.1. Overview

**Figure 5:**
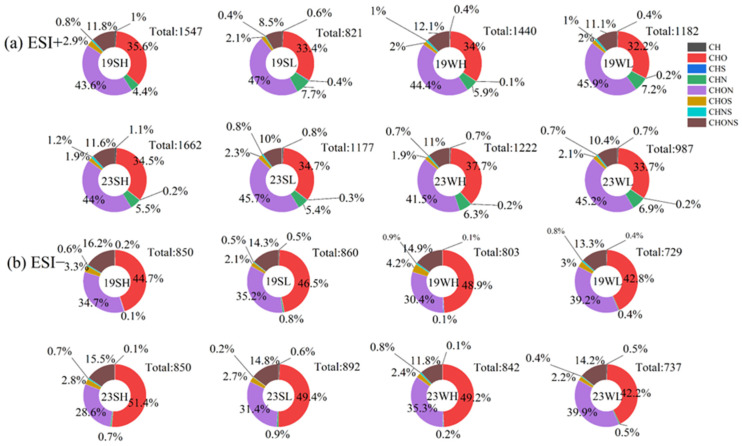
The number fractions of different types of compounds in OA from different periods. 19SH and 19SL represent the selected samples in 2019 summer with relatively high and relatively low PM_2.5_ concentrations, respectively; meanings of 19WH, 19WL, 23SH, 23SL, 23WH and 23WL are similar. (**a**) ESI+ mode; (**b**) ESI− mode.

According to the protocols in Section 2.2, the HPLC-QToF-MS analysis on the methanol extracts identified a total of 729–892 and 821–1662 molecules in negative ion mode (ESI−) and positive ion mode (ESI+), respectively. These molecules were further classified into eight categories: CH, CHO, CHS, CHN, CHON, CHOS, CHNS, and CHONS com-pounds. As shown in Figure 5a, in ESI+ mode, number fraction of CHON species was the largest (41.5~47.0%) in all cases; for samples collected during low-pollution days, its fraction was slightly higher than those during high-pollution days. In terms of relative signal abundance (Figure S4a), CHON remained as the most important group (29.4~44.0%) except for summer 2019. The second most abundant group was CHO, occupying 32.2~37.7% of the total number and 23.4~42.2% of the total signal of identified molecules. Differences in CHO number fractions between summer and winter were insignificant, while those of signal fractions were relatively large, particularly in 2019 (33.9~42.2% in summer vs. 23.4~27.8% in winter). Other relatively important types in number were CHONS (8.5~12.1%) and CHN species (4.4~7.7%); contrastingly, their relatively abundances in signal were only 1.4~4.0% for CHONS but 14.0~35.3% for CHN (Figure 5a and Figure S3a). The rest four compound families were very minor both in number and signal.

In ESI− mode, instead of CHON, CHO species became the most abundant both in number fraction (42.2~51.4%) and signal fraction (38.3~64.6%), and the CHON species became the second (28.6~39.9% in number, and 13.5~43.6% in signal) (Figure 5b and Figure S3b). The high abundance of CHO species in negative mode than in positive ion mode was reported before [51]. A possible cause is that CHO species may include carboxyl compounds (such as organic acids) that are more sensitive in ESI− mode, while organic basic compounds are more sensitive to ESI+ ionization. Additionally, CHOS and CHONS showed higher abundance in ESI− mode than in ESI+ mode, and the CHONS signal fraction even reached ~39% in high-pollution 2019 summer samples (Figure S4b). CHN and the other three types of compounds were almost negligible in ESI+ mode.

Overall differences between 2019 and 2023 samples in ESI+ mode were subtle. In ESI− mode, 2023 summer samples appeared to contain a bit more CHO species but less CHON species than the 2019 summer samples, while on the opposite, 2023 winter samples had more CHON species than the 2019 winter samples. The total number of molecules in summer and winter in 2023 was greater than that in 2019 in both ESI− or ESI+ mode; In ESI− mode, the molecular number in summer 2019 or 2023 was greater than those in winter, while in ESI+ mode, the molecular number in summer 2019 was lower than that in winter, while that in summer 2023 was greater than in winter.

#### 3.3.2. CHO Compounds

**Figure 6:**
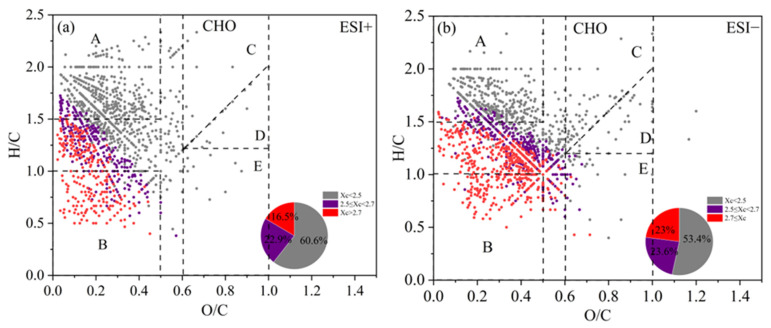
The Van Krevelen (VK) diagram of CHO compounds detected under (**a**) positive ion mode (ESI+) and (**b**) negative ion mode (ESI−) modes. Different colors represent molecules with different aromaticity equivalent (Xc) values, and the pie chart shows corresponding number fractions; dash lines represent the boundaries of difference regimes as explained in the main text.

The Van Krevelen (VK) diagram can distribute the compounds in the H/C vs. O/C space, and the ones for CHO compounds are shown in Figure 6 for both ESI+ and ESI− modes (separate figures for different periods of 2019 and 2023 are presented in Figures S5 and S6). The VK plots can be further divided into five regions [39,84,85,86]: A—Aliphatic compounds (H/C ≥ 1.5, O/C ≤ 0.5); B—Less oxygenated aromatic compounds (H/C ≤ 1.0, O/C ≤ 0.5); C—High oxygenated functional group compounds, such as alcohols, esters, and peroxides (OSc ≥ 0, O/C ≥ 0.6); D—High reduced functional group compounds, such as carbonyls and organic acids (OSc < 0, O/C ≥ 0.6); E—Moderate oxygenated compounds (OSc ≥ 0, O/C ≥ 0, H/C ≤ 1.2). The Xc value of each compound was also calculated and colored in the VK plot. From Figure 6, we can observe that a majority of molecules fall in the regime of O/C < 0.5 (especially regions A and B), but with a wide range of H/C values. Saturated aliphatic CHO species (Xc < 2.5) was the most abundant class of compounds (60.6% and 53.5% of total molecules in ESI+ and ESI− modes), which mainly distributed in region A, with small portions in other regions. Unsaturated CHO compounds with 2.5 ≤ X < 2.7 occupied 22.9% and 23.6%, and those with Xc ≥ 2.7 occupied the remaining 16.5% and 22.5% of the total number of molecules in ESI+ and ESI− modes. These species mainly distributed in region B and that between A and B (Figure 6). Overall, unsaturated compounds were more enriched in ESI− mode than in ESI+ mode. In addition, the aromatic and polycyclic aromatic compounds in both high-pollution and light-pollution samples in winter 2019 were significantly higher than those in summer 2019. However, such difference was not very evident in 2023.

#### 3.3.3. CHON and CHN Compounds

**Figure 7:**
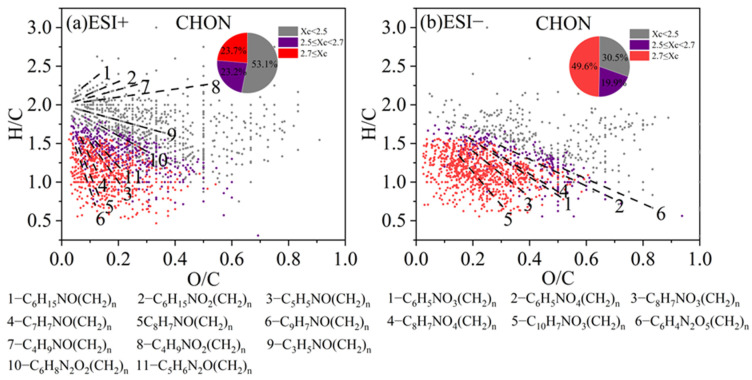
The Van Krevelen (VK) diagram of CHON compounds detected under (**a**) positive ion mode (ESI+) and (**b**) negative ion mode (ESI−) modes. Molecules are also colored according to the aromaticity equivalents (Xc) values and the pie chart shows corresponding number fractions. The different dashed lines represent different series of homologous compounds.

A similar VK diagram for the CHON compounds in both ESI+ and ESI− modes, with colored Xc, is shown in Figure 7. Furthermore, we also classified some species into different series of compounds. In ESI+ mode, CHON compounds containing one -NO functional group dominate the spectrum. CHON compounds were also predominated by the saturated species (Xc < 2.5) (53.1% of total), and their nitrogen-containing functional groups are likely to be of a reduced nature rather than the nitro- or nitroso- groups. For example, a series of organic homologs, such as C_6_H_15_NO(CH_2_)_n_ (likely N,N-diethyl ethanolamine homologs) and C_6_H_15_NO_2_(CH_2_)_n_ (likely diisopropylamine homologs), are likely amines with hydroxyl or ether groups (-O-) and they possess lone pairs of electrons that are prone to protonation [87]. Unsaturated CHON compounds with Xc ≥ 2.5 concentrate in the lower left corner of the plot. Such species include C_9_H_7_NO(CH_2_)_n_, C_8_H_7_NO(CH_2_)_n_, C_7_H_7_NO(CH_2_)_n_, and C_5_H_5_NO(CH_2_)_n_ (lines 3–6 in Figure 7a), possibly corresponding to homologs of hydroxyquinoline, 4-hydroxyphenylethyl nitrile, benzamide, and hydroxy-pyridine, respectively [88,89].

In ESI− mode, the CHON compounds showed a broad distribution. Most compounds contained one or two nitrogen atoms, and instead, a majority (69.5%) of the species were categorized as aromatic compounds with Xc ≥ 2.5, including monocyclic and polycyclic structures. Monocyclic compounds such as C_6_H_5_NO_3_(CH_2_)_n_ and C_6_H_5_NO_4_(CH_2_)_n_ (lines 1–2 in Figure 7b) likely indicate nitrophenol and nitroresorcinol homologs, while C_8_H_7_NO_3_(CH_2_)_n_ and C_8_H_7_NO_4_(CH_2_)_n_ (lines 3–4) may correspond to nitroacetophenone and nitrophenylacetic acid homologs, respectively [28,90,91,92]. Polycyclic CHON compounds, such as C_10_H_7_NO_3_(CH_2_)_n_ (line 5 in Figure 7b), probably refers to nitronaphthol homologs. Moreover, in both ESI+ and ESI− modes, the signal and number fractions of CHON compounds in low-pollution samples are nearly equal or even higher than those in high-pollution samples. This finding aligns with previous studies [89,90,93], and together with the fact that CHON species contains many nitro-aromatic species may suggest that the CHON species are related to various secondary reactions such as photochemical aging.

**Figure 8:**
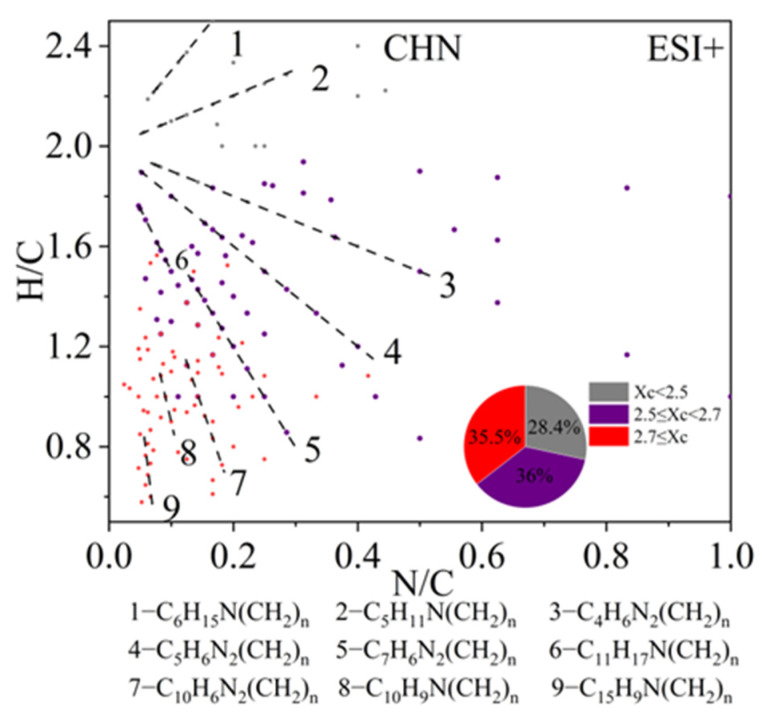
The H/C versus N/C plot of CHN compounds detected under positive ion mode. Molecules are also colored by the aromaticity equivalents (Xc) values and the pie chart shows corresponding number fractions. Different dashed lines represent different series of homologous compounds.

CHN compounds are only enriched in ESI+ mode. The scatter plot in Figure 8 shows the H/C versus N/C ratios for all CHN compounds detected in ESI+ mode. Most of the CHN compounds were amines containing one or two nitrogen atoms. Saturated amines and other monocyclic species (Xc < 2.7) distributed in the upper-middle region of the plot, including C_6_H_15_N(CH_2_)_n_, C_5_H_11_N(CH_2_)_n_, C_4_H_6_N_2_(CH_2_)_n_, C_5_H_6_N_2_(CH_2_)_n_, C_7_H_6_N_2_(CH_2_)_n_, and C_11_H_17_N(CH_2_)_n_ (lines 1–6 in Figure 8). Furthermore, many studies have found that species containing two-membered heterocyclic structures are important components of biomass burning OA [94]. The homologs C_4_H_6_N_2_(CH_2_)_n_, C_5_H_6_N_2_(CH_2_)_n_, and C_7_H_6_N_2_(CH_2_)_n_ are believed to be potential markers for biomass burning. In the lower-left corner of the plot, some CHN compounds with Xc > 2.7 were observed, including a series of PAHs) containing one nitrogen atom, such as C_10_H_9_N(CH_2_)_n_ and C_15_H_9_N(CH_2_)_n_. C_10_H_9_N(CH_2_)_n_ is likely an amino-naphthalene homolog [87], which may originate from the combustion of carbonaceous materials. Previous studies have also suggested that 1N-PAHs can be used as markers of coal combustion [37].

#### 3.3.4. CHOS and CHONS Compounds

**Figure 9:**
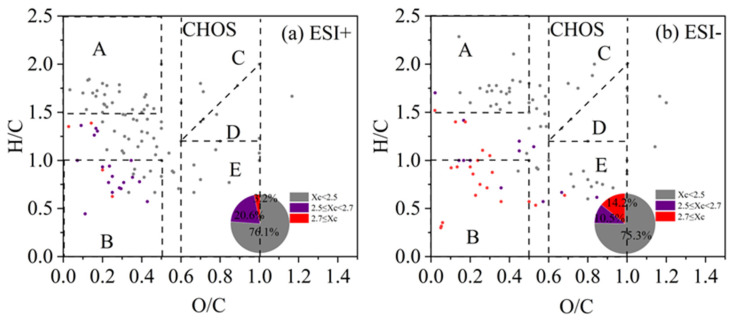
The Van Krevelen (VK) diagram of CHOS compounds detected under (**a**) positive ion mode (ESI+) and (**b**) negative ion mode (ESI−) modes. Molecules are colored according to the aromaticity equivalents (Xc) values and the pie chart shows corresponding number fractions.

**Figure 10:**
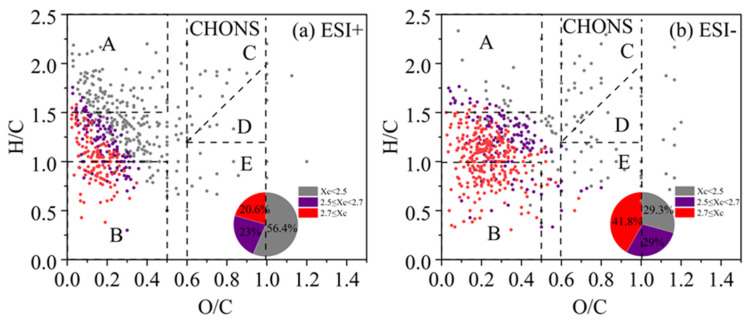
The Van Krevelen (VK) diagram of CHONS compounds detected under (**a**) positive ion mode (ESI+) and (**b**) negative ion mode (ESI−) modes. Molecules are colored according to the aromatici-ty equivalents (Xc) values and the pie chart shows corresponding number fractions.

The VK plots for CHOS and CHONS compounds are illustrated in Figure 9 and Figure 10, respectively. The majority of CHOS compounds were composed of saturated aliphatic compounds (Xc < 2.5) (75.3% and 76.1% in ESI− and ESI+ modes, respectively). Relatively more CHOS compounds resided in the regime of O/C < 0.5, and number fraction of compounds with 2.5 < Xc ≤ 2.7 was also notably higher in ESI+ mode than in ESI− mode. For the CHONS compounds, number fraction of aliphatic species (Xc < 2.5) was still prevailing (56.4%) (contributions of the other two types were on par, 23.0% and 20.6%, respectively) in ESI+ mode, while in ESI− mode, compounds with Xc > 2.7 (polycyclic aromatic species) became the most important portion (41.8%), and fraction of aliphatic species was down to only 29.3%. CHONS species behaved quite distinctly in positive and negative modes and its signal fraction in ESI+ model was quite low relative to the number fraction (Figure S4).

### 3.4. Characteristics of OA Molecules

#### 3.4.1. Tracer Compounds of Different Sources

**Figure 11:**
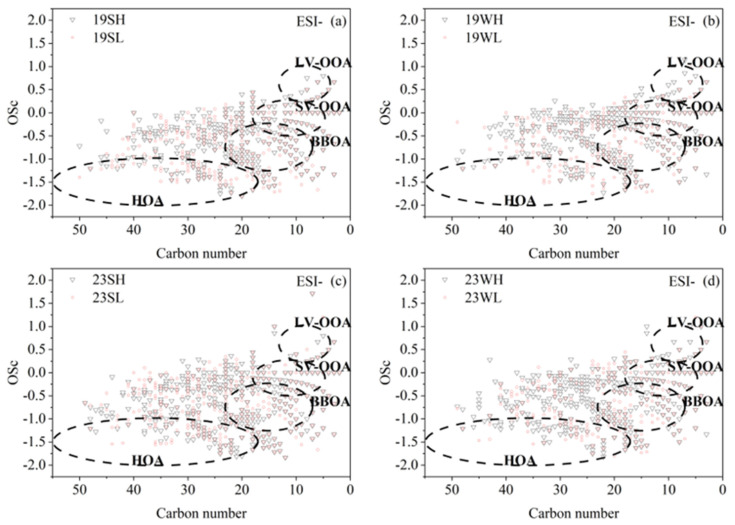
The carbon oxidation states (OSc) of CHO compounds against carbon numbers of the compounds. The black dashed circled regions are labeled as hydrocarbon-like OA (HOA), biomass burning OA (BBOA), semi-volatile oxygenated OA (SV-OOA), and low-volatility oxygenated OA (LV-OOA) (Kroll et al., 2011 [50]). 19SH and 19SL represent the selected samples in 2019 summer with relatively high and relatively low PM_2.5_ concentrations, respectively; meanings of 19WH, 19WL, 23SH, 23SL, 23WH and 23WL are similar. Results of (**a**) 2019 summer, (**b**) 2019 winter, (**c**) 2023 summer, (**d**) 2023 winter.

The ambient OA can contain molecules from multiple primary sources and various secondary processes, which are affected by meteorological conditions, transportation, chemical reactions, deposition, etc. The OSc value can be used to assess the aging or oxidation degree of OA and infer their potential sources [50]. For the most abundant CHO compounds, Figure 11 and Figure S7 demonstrate the relationships between the OSc and the number of carbon atoms for CHO compounds of both ESI+ and ESI− modes, and the data points are roughly assigned to different sources based on results from a previous study [50]: HOA (hydrocarbon-like OA, associated with fossil fuel combustion such as traffic); BBOA (biomass burning OA); SV-OOA (semi-volatile oxygenated OA, secondarily formed generally with a low oxidation degree); LV-OOA (low-volatility oxygenated OA, secondarily formed generally with a high oxidation degree). As presented, a significant number of compounds were categorized as HOA and BBOA in both ESI− and ESI+ modes, reflecting the large impact of anthropogenic combustion activities on OA in Nanjing; the number of BBOA species in ESI− mode were also significantly more than those in ESI+ mode, and the HOA and BBOA species were also a bit more in 2023 than in 2019. A number of species were also identified as potential SV-OOA but much less species belonged to LV-OOA, in agreement with the overall low O/C of CHO compounds shown in Figure 6, indicating that OA in Nanjing was overall relatively fresh.

Since non-targeted molecular characterization is inherently good in identification rather than quantification of the OA compounds, hereby we only selected a few molecules with relatively large signals representing different sources to examine the OA properties in different periods (Table S4). For LV-OOA, SV-OOA and HOA, we chose the compounds locating in the regimes marked in Figure 11. For LV-OOA, we selected C_5_H_8_O_5_ and C_5_H_6_O_4_, and for SV-OOA, we selected C_6_H_10_O_5_ and C_14_H_16_O_8_. Signal fractions of these four molecules were slightly higher in summer than in winter, suggesting that their secondary formations were likely more important in summer. Another finding is that their overall in-fluences on OA were also higher in high-pollution samples, indicating that secondary re-actions became even more important during high-pollution episodes. For HOA, we selected C_27_H_44_O_6_, and its portion was higher in winter than in summer, whose value in 2023 decreased from that of 2019. The result is reasonable as fossil fuel combustion emissions are typically more significant in winter, while the contribution might be reduced due to emission control in 2023 relative to that in 2019. For the biomass burning OA (BBOA), we chose C_6_H_10_O_5_, C_9_H_8_O_3_, and C_6_H_5_NO_4_, likely levoglucosan, pentose aldehyde (levoglucosan aldehyde) (derived from lignin decomposition), and 4-nitrocatechol, respectively [95,96,97]. Signal proportions of these BBOA tracers in winter were notably higher than those observed in summer; their proportions were larger in low-pollution samples in 2019, while they were larger in high-pollution samples in 2023, suggesting a change of BBOA’s role in the PM_2.5_ pollution in Nanjing. For cooking OA (COA) [98], we selected C_16_H_30_O_3_ and C_18_H_34_O_3_, and their signal proportions were found to be higher in summer and in high pollution samples.

Furthermore, several secondary acids, including C_7_H_10_O_4_, C_8_H_12_O_4_, C_10_H_16_O_3_, C_10_H_16_O_4_, C_8_H_12_O_6_, and C_10_H_16_O_6_, were identified in our samples. These compounds, previously confirmed as photochemically-derived SOA species from α-pinene oxidation [99], exhibited higher proportions in summer (0.495%) than in winter (0.203%), emphasizing the role of photo-oxidation in summer (Table S5). Their summer average increased from 0.437% in 2019 to 0.558% in 2023, suggesting an enhanced photochemical influence after the pandemic. We also examined three aqueous-phase SOA (AqSOA) markers [20,100], such as pyruvic acid (C_3_H_4_O_3_), oxalic acid (C_2_H_2_O_4_), and dimethyl nitrobenzoic acid (C_9_H_9_NO_4_). Their average proportion was lower in summer (0.087%) than in winter (0.189%), indicating a greater role of aqueous-phase reactions in winter. Furthermore, phenolic derivatives, key contributors to aqueous-phase SOA, originate from lignin pyrolysis and are closely linked to biomass burning [101]. As shown in the Table S6, phenolic-derived oligomers were more abundant in winter, further highlighting the significance of aqueous-phase reactions and BBOA contributions to OA during this season.

At last, organosulfates (OrgSs) are a prominent class of CHOS species, typically having oxygen-to-sulfur (O/S) ratios ≥ 4. As listed in Table 3, in -ESI− mode, the number proportion of OSs (on average 70.8%) was slightly higher than that in ESI+ mode (average 64.6%); and the fraction was higher in 2019 winter than in 2023 winter in ESI+ mode, but differences in other periods were not significant. For CHONS compounds, 5.1–17.4% of the molecules in ESI− mode (signals in ESI+ mode were very low, therefore were not considered here) can be assigned to nitrooxy-OrgSs (with an O/(4S+3N) ratio ≥ 1, and possibly -OSO_3_H and -ONO_2_ functional groups) [38]; there was a relatively large difference between high- and low-pollution samples—higher fraction in low-pollution samples, which is consistent with a previous study [38]. Song et al. [102] point out that coal combustion may be an important sources of S-containing compounds, while biomass burning is a source of atmospheric N-containing compounds, which can further form nitroaromatic compounds upon atmospheric aging [103]. Some previous studies also indicate that biogenic, motor vehicular and ship emissions, etc. might promote the formation of Nitro-OrgSs too [104,105,106,107]. However, the nitro-oxy-OrgSs are mostly likel associated with aqueous phase reactions [108]. Since their relative fractions were high in low-pollution samples closely associated with high RH conditions in this work (Table S3), suggesting aqueous oxidation might be the most likely source of such species.

#### 3.4.2. Correlations with Light Absorption

To investigate the link between optical properties and molecular composition, we investigated the correlations between the signal fractions of different types of molecules and the MAE_MSOC-365_ values, as shown in Table 4. Very interestingly, correlations of CH, CHO, CHN, CHON and CHS in ESI− mode with MAE_MSOC-365_ during summer were positive, while the correlations became negative (except CHO and CHS) during winter; on the contrary, correlations of CHOS, CHNS and CHONS in ESI− mode with MAE_MSOC-365_ were all negative during summer but all positive during winter. In ESI+ mode, except that correlations of CH and CHN with MAE_MSOC-365_ were all positive in both summer and winter, the correlation coefficients of CHO, CHS, CHON, CHOS, CHNS and CHONS in summer were all appositive to those in winter. These results likely suggest a big compositional difference of the key light absorbing molecules between summer and winter. Furthermore, in summer, MAE_365-MSOC_ correlated fairly well with CH, CHS, CHN, and CHON in ESI− mode (*r* > 0.5) and with CH and CHN in ESI+ mode (*r* > 0.5). In winter, it showed relatively good correlations with CHO, CHS, CHOS (*r* > 0.5) and CHNS (*r* of 0.46) in ESI− mode, and with CHN (*r* of 0.82) and CHNS (*r* of 0.97) in ESI+ mode. Overall, CH and CHN compounds contributed significantly to light absorption in summer, while the role of CHNS compounds became a bit more important in winter.

## 4. Conclusions

In this work, we conducted a comprehensive analysis on the optical properties and molecular chemical composition of OA in Nanjing by using samples collected in summer and winter of 2019 and 2023. The results show a clear decline of EC, OC, MSOC and WSOC concentrations from 2019 to 2023 in both summer and winter; in general, Abs_365_ and MAE_365_ values for both WSOC and MSOC were higher in winter than in summer likely due to increased combustion activities, and those of MSOC were higher than MSOC in all periods. Fluorescent properties of both WSOC and MSOC were dominated by humic-like components but the remaining contribution from protein-like component was more significant in MSOC. Fluorescence indices suggest that OA in Nanjing was influenced by a mix of microbial/biogenic source. In particular, the HIX values in 2023 for both WSOC and MSOC appeared to drop significantly from those in 2019, demonstrating a big decrease of OA’s aromaticity in Nanjing.

Results of OA molecular composition did not show remarkable difference between 2019 and 2023. Overall, in term of number of identified molecules, the CHON compounds were the most abundant type of species, followed by the CHO and CHN compounds; aliphatic compounds dominated the major molecular types except for CHN (in ESI+ mode) and CHON, CHOS (in ESI− mode). Qualitative source analysis shows the numbers of molecules from fossil fuel combustion and biomass burning (BB) were a bit more in 2023 than in 2019, and signal intensities of BB-related molecules were also higher in winter than in summer. The presence of organosulfates further indicates the contribution of aqueous-phase oxidation to OA, especially during high relative humidity conditions; identification of a series of phenolic species and their oxidation products also suggests that BB emitted species might be precursors of the aqueous-phase oxidation. At last, cross-correlations between OA molecules (in signal fractions) and light absorption (in MAE) indicate that the key light-absorbing species in winter and summer were likely quite different despite their similar chemical compositions. In summer, CH and CHN compounds were important to light absorption, whereas CHNS compounds became more important in winter. Our findings regarding the optical and chemical properties of ambient OA here are implicate for future PM2.5 reduction and air quality improvement.

## Figures and Tables

**Table 1:** Mass concentrations (±one standard deviation) and optical parameters of carbonaceous components at different sampling periods in Nanjing, China.

Terms	2019		2023	
	Summer (*n* = 73)	Winter (*n* = 38)	Summer (*n* = 52)	Winter (*n* = 30)
EC (μg m^−3^)	1.17 ± 0.34	1.72 ± 1.08	0.94 ± 0.43	1.02 ± 0.79
OC (μg m^−3^)	8.73 ± 2.49	12.39 ± 5.06	6.70 ± 2.82	10.25 ± 6.11
WSOC (μg m^−3^)	5.34 ± 1.59	6.73 ± 2.74	3.95 ± 1.75	5.69 ± 2.96
MSOC (μg m^−3^)	7.25 ± 2.21	10.94 ± 4.65	6.06 ± 2.71	8.26 ± 4.85
Water-soluble OC (WSOC)				
Abs_WSOC-365_ (M m^−1^)	2.87 ± 0.86	5.70 ± 3.56	1.76 ± 1.00	3.73 ± 2.46
MAE_WSOC-365_ (m^2^ g^−1^)	0.54 ± 0.14	0.81 ± 0.29	0.45 ±0.15	0.62 ± 0.18
AAE_WSOC_	6.04 ± 0.40	6.64 ± 1.21	6.28 ± 0.61	6.53 ± 0.71
k_WSOC-365_	0.0236 ± 0.0060	0.0355 ± 0.0127	0.0195 ± 0.0066	0.0271 ± 0.0078
Methanol-soluble OC (MSOC)				
Abs_MSOC-365_ (M m^−1^)	3.92 ± 0.95	12.73 ± 4.56	2.97 ± 1.12	5.14 ± 2.65
MAE_MSOC-365_ (m^2^ g^−1^)	0.57 ± 0.19	1.20 ± 0.23	0.52 ± 0.15	0.65 ± 0.15
AAE_MSOC_	5.53 ± 0.50	4.40 ± 0.32	6.34 ± 0.96	6.09 ± 0.32
*k*_MSOC__-__365_	0.0247 ± 0.0085	0.0525 ± 0.0099	0.0226 ± 0.0065	0.0284 ± 0.0064

**Table 2:** Average Fluorescence Index Values of Water-Soluble Organic Carbon (WSOC) and Methanol-Soluble Organic Carbon (MSOC).

	2019		2023	
	Summer	Winter	Summer	Winter
WSOC				
FI_WSOC_	1.91 ± 0.18	1.87 ± 0.07	2.02 ± 0.26	1.91 ± 0.09
BIX_WSOC_	0.86 ± 0.08	0.87 ± 0.09	0.85 ± 0.09	0.86 ± 0.06
HIX_WSOC_	5.58 ± 1.07	7.16 ± 1.06	5.24 ± 0.70	6.09 ± 0.77
MSOC				
FI_MSOC_	2.62 ± 0.44	2.70 ± 0.28	3.35 ± 0.62	2.25 ± 0.18
BIX_MSOC_	0.93 ± 0.10	1.00 ± 0.10	0.85 ± 0.14	1.04 ± 0.13
HIX_MSOC_	3.34 ± 0.81	3.87 ± 0.43	2.80 ± 0.62	3.05 ± 0.64

**Table 3:** The number percentages of the organosulfates (OrgSs) in CHOS compounds under two modes and those of nitrooxy-OrgSs in CHONS compounds under ESI− mode.

		2019 Summer		2019 Winter		2023 Summer		2023 Winter	
		2019SH	2019SL	2019WH	2019WL	2023SH	2023SL	2023WH	2023WL
CHOS	OrgSs (ESI−)	75.0%	77.8%	64.7%	86.4%	75.0%	70.8%	60.0%	56.3%
	OrgSs (ESI+)	68.9%	70.6%	62.1%	66.7%	62.5%	63.0%	60.9%	61.9%
CHNOS	Nitrooxy-OrgSs (ESI−)	9.4%	16.3%	15.0%	16.5%	10.6%	17.4%	5.1%	14.3%

**Table 4:** The cross-correlation coefficients (Pearson’s *r*) between different types of OA molecules (relative signal intensities) with MAE_MSOC-365_ in summer and winter (2019 and 2023 samples combined).

Mode	Type	Summer	Winter
		MAE_MSOC-365_	MAE_MSOC-365_
ESI−	CH	0.78	−0.15
	CHO	0.23	0.59
	CHS	0.76	0.66
	CHN	0.81	−0.7
	CHON	0.5	−0.68
	CHOS	−0.71	0.83
	CHNS	−0.49	0.46
	CHONS	−0.43	0.35
ESI+	CH	0.66	0.39
	CHO	0.38	−0.87
	CHS	0.42	−0.59
	CHN	0.69	0.82
	CHON	0.32	−0.41
	CHOS	0.48	−0.94
	CHNS	−0.44	0.97
	CHONS	0.49	−0.92

## Data Availability

The data presented in this article are available on request from the corresponding author.

## Supplementary Materials

The following supporting information can be downloaded at https://www.mdpi.com/article/10.3390/toxics13060443/s1, Table S1: The test results of normality distribution of the mass concentrations and optical parameters of samples during different periods; Table S2: The Wilcoxon significance test results of mass concentrations and relevant optical parameter of different pairs of sampling periods; Table S3: The relative humidity and temperature of the selected 24 samples for OA molecular characterization; Table S4: The relative signal fractions of the selected HOA, BBOA, SV-OOA, and LV-OOA tracer compounds in the samples; Table S5: The relative signal fractions of some selected SOA tracer molecules in the samples; Table S6: The relative signal fractions of some oligomers derived from aqueous-oxidation of phenolic substances (phenol, syringol and eugenol) in the samples; Figure S1: The sample TIC and mass spectra of ESI+ and ESI−; Figure S2: The correlation plots (Pearson’s r) of: (**a**) water-soluble organic carbon (WSOC) versus its light absorption at 365 nm (Abs_WSOC-365_); (**b**) methanol-soluble organic carbon (MSOC) versus its light absorption at 365 nm (Abs_MSOC-365_); (**c**) WSOC versus MSOC; (**d**) Abs_WSOC-365_ versus Abs_MSOC-365_; Figure S3: The relative signal fractions of the resolved fluorescent components derived from parallel factor analysis (PARAFAC) for (**a**) water-soluble organic carbon (WSOC) and (**b**) methanol-soluble organic carbon (MSOC) during different sampling periods; Figure S4: The relative signal fractions of different types of compounds in OA from different periods; Figure S5: The Van Krevelen (VK) diagram of CHO compounds detected under ESI+ mode; Figure S6: The Van Krevelen (VK) diagram of CHO compounds detected under ESI− mode; Figure S7: Carbon oxidation state (OSc) diagram of CHO compounds under ESI+ mode.
