# Supplementary material for: Optical Properties and Molecular Composition of Fine Organic Aerosols in Nanjing, China: A Comparison of 2019 and 2023
Source: Toxics. 2025 May 27;13(6):443. doi: 10.3390/toxics13060443 (PMC12197671; doi:10.3390/toxics13060443)

Toxics

Supplementary Materials: Optical Properties and Molecular Composition of Fine Organic Aerosols in Nanjing, China: A comparison of 2019 and 2023

Table S1. The test results of normality distribution (Kolmogorov-Smirnova or Shapiro-Wilk tests) of the mass concentrations and optical parameters of samples during different periods (the value larger than 0.05) indicates a normality distribution and those less than 0.05 underlined indicate non-normality distribution; values less than 0.001 are written as 0.000)

Normal distribution significance value	2019SUM	2019WIN	2023SUM	2023WIN
EC (μg m^{-3})	0.024	0.000	0.027	0.000
OC (μg m^{-3})	0.200	0.094	0.670	0.000
WSOC (μg m^{-3})	0.200	0.521	0.038	0.041
MSOC (μg m^{-3})	0.200	0.059	0.242	0.002
Abs$_{WSOC-365}$ (M m^{-1})	0.200	0.000	0.000	0.012
MAE$_{WSOC-365}$ (m^2 g^{-1})	0.200	0.000	0.000	0.517
Abs$_{MSOC-365}$ (M m^{-1})	0.200	0.657	0.099	0.026
MAE$_{MSOC-365}$ (m^2 g^{-1})	0.130	0.020	0.200	0.948
k$_{WSOC-365}$	0.215	0.000	0.010	0.569
k$_{MSOC-365}$	0.200	0.127	0.200	0.946
AAE$_{WSOC}$	0.050	0.000	0.200	0.322
AAE$_{MSOC}$	0.200	0.220	0.200	0.508

Table S2. The Wilcoxon significance test results of mass concentrations and relevant optical parameter of different pairs of sampling periods (hypothesis testing *p* values on 95% confidence interval (the result less than 0.001 are written as 0.000)

p value	2019SUM VS 2019WIN	2019SUM VS 2023SUM	2019WIN VS 2023WIN	2023SUM VS 2023WIN
EC ($\mu g\ m^{-3}$)	0.000	0.006	0.006	0.010
OC ($\mu g\ m^{-3}$)	0.003	0.000	0.000	0.041
WSOC ($\mu g\ m^{-3}$)	0.049	0.000	0.028	0.041
MSOC ($\mu g\ m^{-3}$)	0.003	0.001	0.032	0.029
$Abs_{WSOC\text{-}365}$ ($M\ m^{-1}$)	0.000	0.000	0.036	0.002
$MAE_{WSOC\text{-}365}$ ($m^2\ g^{-1}$)	0.000	0.000	0.000	0.001
$Abs_{MSOC\text{-}365}$ ($M\ m^{-1}$)	0.000	0.000	0.000	0.000
$MAE_{MSOC\text{-}365}$ ($m^2\ g^{-1}$)	0.000	0.010	0.000	0.002
$k_{WSOC\text{-}365}$	0.000	0.000	0.000	0.001
$k_{MSOC\text{-}365}$	0.000	0.034	0.000	0.005
AAE_{WSOC}	0.008	0.035	0.024	0.004
AAE_{MSOC}	0.000	0.000	0.000	0.026

Table S3. The relative humidity and temperature of the selected 24 samples for OA molecular characterization (19SH and 19SL represent the selected samples in 2019 summer with relatively high and relatively low PM2.5 concentrations, respectively; meanings of 19WH, 19WL, 23SH, 23SL, 23WH and 23WL are similar)

.		Sample ID	T (℃)	RH (%)
19Sunmmer	19SH	Sample1	25.52	51.60
		Sample2	27.87	50.88
		Sample3	28.95	51.59
	19SL	Sample4	26.81	56.82
		Sample5	27.76	75.95
		Sample6	30.90	65.30
19Winter	19WH	Sample7	7.22	52.71
		Sample8	9.71	48.04
		Sample9	11.22	55.57
	19WL	Sample10	6.48	56.81
		Sample11	7.19	72.60
		Sample12	10.03	83.75
23Sunmmer	23SH	Sample13	28.43	49.86
		Sample14	28.96	80.76
		Sample15	30.21	76.33
	23SL	Sample16	28.84	76.82
		Sample17	28.37	81.72
		Sample18	30.06	77.03
23Winter	23WH	Sample19	12.65	41.03
		Sample20	6.14	67.54
		Sample21	8.94	80.74
	23WL	Sample22	-2.06	40.06
		Sample23	6.50	63.48
		Sample24	4.97	88.82

Table S4. The relative signal fractions of the selected HOA, BBOA, SV-OOA, and LV-OOA tracer compounds in the samples (meanings of 19SH, 19SL, 19WH, 19WL, 23SH, 23SL, 23WH, and 23WL are the same as those in Table S1).

	LV-OOA		SV-OOA		BBOA			COA		HOA
	$C_5H_8O_5$	$C_5H_6O_4$	$C_6H_{10}O_5$	$C_{14}H_{16}O_8$	$C_6H_{10}O_5$	$C_9H_8O_3$	$C_6H_5NO_4$	$C_{16}H_{30}O_3$	$C_{18}H_{34}O_3$	$C_{27}H_{44}O_6$
	0.649%	0.302%	0.571%	0.304%	0.571%		0.436%	0.079%		0.026%
19SH	0.563%	0.302%	0.452%	0.298%	0.452%		0.325%	0.082%		0.025%
	0.549%	0.260%	0.448%	0.276%	0.448%		0.412%	0.054%		0.025%
	0.832%	0.527%	0.651%	0.274%	0.651%	0.175%	0.162%	0.082%		0.037%
19SL	0.701%	0.808%	0.856%	0.198%	0.856%	0.194%	0.666%	0.073%		0.019%
	0.445%	0.479%	0.630%	0.244%	0.630%	0.136%	1.067%	0.127%		0.042%
	0.936%	0.433%	0.355%	0.309%	0.355%	0.345%	2.210%	0.036%	0.017%	0.055%
19WH	0.677%	0.348%	0.421%	0.143%	0.421%	0.440%	1.413%	0.037%	0.015%	0.029%
	0.575%	0.272%	0.251%	0.395%	0.251%	0.090%	4.870%	0.047%	0.015%	0.047%
		0.163%		0.130%		0.376%	12.830%	0.065%	0.148%	0.044%
19WL		0.553%		0.238%		0.469%	4.344%	0.072%	0.074%	0.032%
		0.816%		0.236%		0.613%	6.146%	0.096%	0.250%	0.054%
	0.484%	0.285%	0.459%	0.292%	0.459%		0.800%	0.066%		0.016%
23SH	0.622%	0.288%	0.548%	0.268%	0.548%		0.305%	0.042%		0.007%
	0.566%	0.413%	0.372%	0.183%	0.372%		0.130%	0.078%		0.005%
	0.306%	0.371%	0.473%	0.164%	0.473%	0.101%		0.107%		0.035%
23SL	0.421%	0.281%	0.351%	0.225%	0.351%	0.076%		0.081%		0.019%
	0.250%	0.216%	0.397%	0.196%	0.397%	0.072%		0.092%		0.018%
	1.057%	0.185%		0.055%			7.860%	0.143%	0.084%	0.040%
23WH	0.616%	0.096%		0.141%			5.345%	0.035%	0.011%	0.047%
	0.825%	0.323%		0.495%			0.920%	0.045%	0.014%	0.018%
		0.250%		0.100%		0.087%	0.957%	0.055%	0.047%	0.036%
23WL		0.244%		0.155%		0.184%	2.577%	0.061%	0.032%	0.024%
		0.731%		0.230%		0.266%	0.805%	0.072%	0.023%	0.036%

Table S5. The relative signal fractions of some selected SOA tracer molecules in the samples (meanings of 19SH, 19SL, 19WH, 19WL, 23SH, 23SL, 23WH, and 23WL are the same as those in Table S1).

	PtSOA						AqSOA		
	$C_7H_{10}O_4$	$C_8H_{12}O_4$	$C_{10}H_{16}O_3$	$C_{10}H_{16}O_4$	$C_8H_{12}O_6$	$C_{10}H_{16}O_6$	$C_3H_4O_3$	$C_3H_4O_2$	$C_9H_9NO_4$
19SH	0.287%	0.384%	0.148%	0.179%	0.721%	0.369%	0.125%	0.019%	0.042%
	0.280%	0.426%	0.141%	0.243%	0.656%	0.130%	0.114%	0.018%	0.031%
	0.262%	0.393%	0.113%	0.177%	0.719%	0.389%	0.084%	0.013%	0.039%
19SL	0.576%	0.913%	0.175%	0.233%	1.761%	0.719%	0.074%	0.042%	0.132%
	0.563%	0.616%	0.187%	0.330%	0.871%	0.383%	0.185%	0.112%	0.209%
	0.364%	0.611%	0.163%	0.346%	0.504%	0.387%	0.158%	0.056%	0.210%
19WH	0.246%	0.090%	0.024%	0.124%	0.337%	0.193%	0.031%	0.011%	0.158%
	0.199%	0.080%	0.024%	0.124%	0.280%	0.160%	0.106%	0.036%	0.200%
	0.173%	0.066%	0.022%	0.114%	0.386%	0.158%	0.071%	0.011%	0.298%
19WL	0.200%	0.280%	0.078%	0.067%	0.189%	0.196%	0.110%	0.037%	0.346%
	0.208%	0.351%	0.070%	0.062%	0.355%	0.255%	0.112%	0.079%	0.302%
	0.239%	0.406%	0.057%	0.294%	0.227%	0.171%	0.735%	0.135%	0.294%
23SH	0.281%	0.368%	0.159%		0.619%	0.390%	0.136%	0.057%	0.080%
	0.239%	0.368%	0.115%		0.886%	0.531%	0.104%	0.047%	0.073%
	0.874%	2.165%	0.670%		2.149%	0.830%	0.098%	0.125%	0.054%
23SL	0.348%	0.537%	0.222%	0.259%	0.335%	0.295%	0.123%	0.046%	
	0.448%	1.102%	0.151%	0.318%	1.100%	0.577%	0.091%	0.058%	
	0.242%	0.590%	0.154%	0.331%	0.412%	0.342%	0.086%	0.030%	
23WH	0.262%	0.415%	0.473%	0.271%	0.299%	0.160%	0.030%	0.016%	1.099%
	0.232%	0.285%	0.102%	0.116%	0.493%	0.215%	0.178%	0.017%	0.553%
	0.348%	0.503%	0.065%	0.141%	0.551%	0.310%	0.329%	0.021%	0.197%
23WL	0.164%	0.299%	0.134%	0.159%	0.121%	0.062%	0.022%	0.037%	0.104%
	0.144%	0.209%	0.111%	0.172%	0.195%	0.112%	0.030%	0.017%	0.343%
	0.222%	0.210%	0.055%	0.074%	0.233%	0.182%	0.371%	0.179%	0.187%

Table S6. The relative signal fractions of some oligomers derived from aqueous-oxidation of phenolic substances (phenol, syringol and guaiacol) in the samples (meanings of 19SH, 19SL, 19WH, 19WL, 23SH, 23SL, 23WH, and 23WL are the same as those in Table S1).

		phenol	syringol								guaiacol	
		$C_8H_6O_5$	$C_{16}H_{18}O_6$	$C_{15}H_{16}O_6$	$C_{15}H_{18}O_7$	$C_{14}H_{12}O_7$	$C_{13}H_{14}O_7$	$C_{12}H_{12}O_7$	$C_{16}H_{18}O_7$	$C_{14}H_{16}O_9$	$C_{14}H_{12}O_6$	$C_7H_{10}O_6$
		1.107%	0.456%		1.059%					0.544%		1.440%
	19SH	0.917%	0.500%		1.276%					0.437%		1.188%
19Summer		1.015%	0.541%		1.450%					0.421%		1.299%
		0.872%	0.342%		0.797%			1.234%				1.563%
	19SL	1.055%	0.268%		0.800%			0.699%				1.202%
		1.453%	0.367%		1.197%			0.846%				0.727%
		4.361%	0.177%	0.169%	0.403%	0.423%	0.906%	1.594%	0.248%	0.490%	0.432%	0.964%
	19WH	3.500%	0.132%	0.377%	0.831%	0.422%	1.075%	1.385%	0.496%	0.323%	0.121%	0.755%
19Winter		3.878%	0.163%	0.517%	0.469%	0.567%	1.357%	1.654%	0.276%	0.394%	0.124%	0.949%
		2.898%			0.280%			0.959%				0.847%
	19WL	2.915%			0.829%			0.878%				0.908%
		2.497%			0.774%			0.887%				0.630%
			0.344%	0.324%	0.932%							1.085%
	23SH		0.455%	0.330%	1.064%							1.439%
23Summer			0.211%	0.168%	0.420%							1.264%
		0.474%			0.689%							0.441%
	23SL	0.360%			0.483%							0.700%
		0.334%			0.878%							0.342%
		0.612%	0.310%	0.146%	0.336%		0.216%	0.074%	0.403%			0.310%
	23WH	2.099%	0.288%	0.185%	0.645%		0.482%	0.397%	0.419%			0.757%
23Winter		1.878%	0.352%	0.414%	0.621%		1.172%	1.445%	0.291%			1.161%
		0.967%		0.192%	0.230%						0.130%	
	23WL	0.522%		0.239%	0.431%						0.144%	
		1.549%		0.264%	0.419%						0.203%	

Figure S1. (a) The total ion chromatography (TIC) diagram, and corresponding mass spectra of both positive ion (b) and negative ion (c) modes of a typical sample

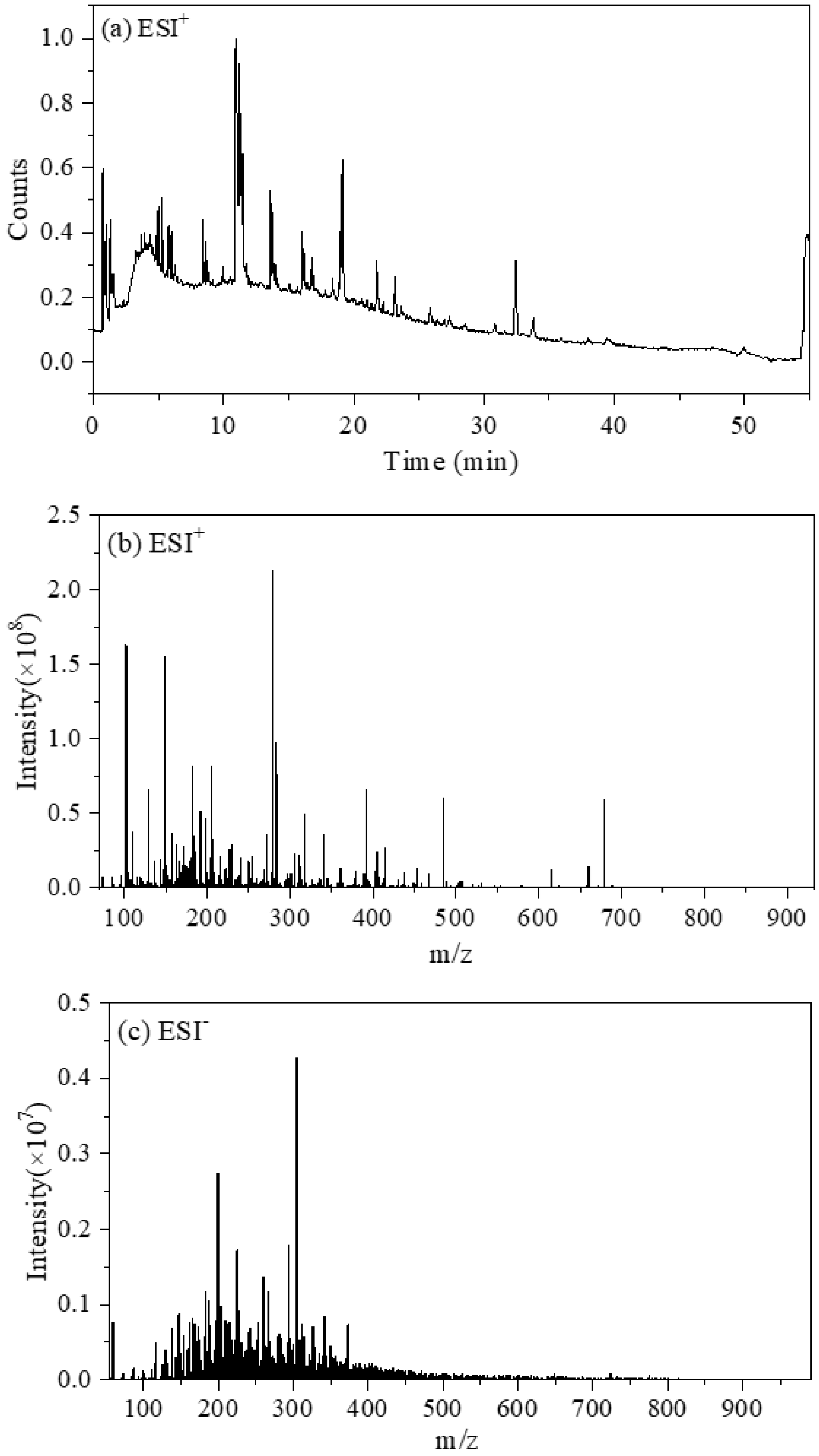

Figure S2. The correlation plots (Pearson's r) of: (a) water-soluble organic carbon (WSOC) *versus* its light absorption at 365 nm ($Abs_{WSOC-365}$); (b) methanol-soluble organic carbon (MSOC) *versus* its light absorption at 365 nm ($Abs_{MSOC-365}$); (c) WSOC *versus* MSOC; (d) $Abs_{WSOC-365}$ *versus* $Abs_{MSOC-365}$.

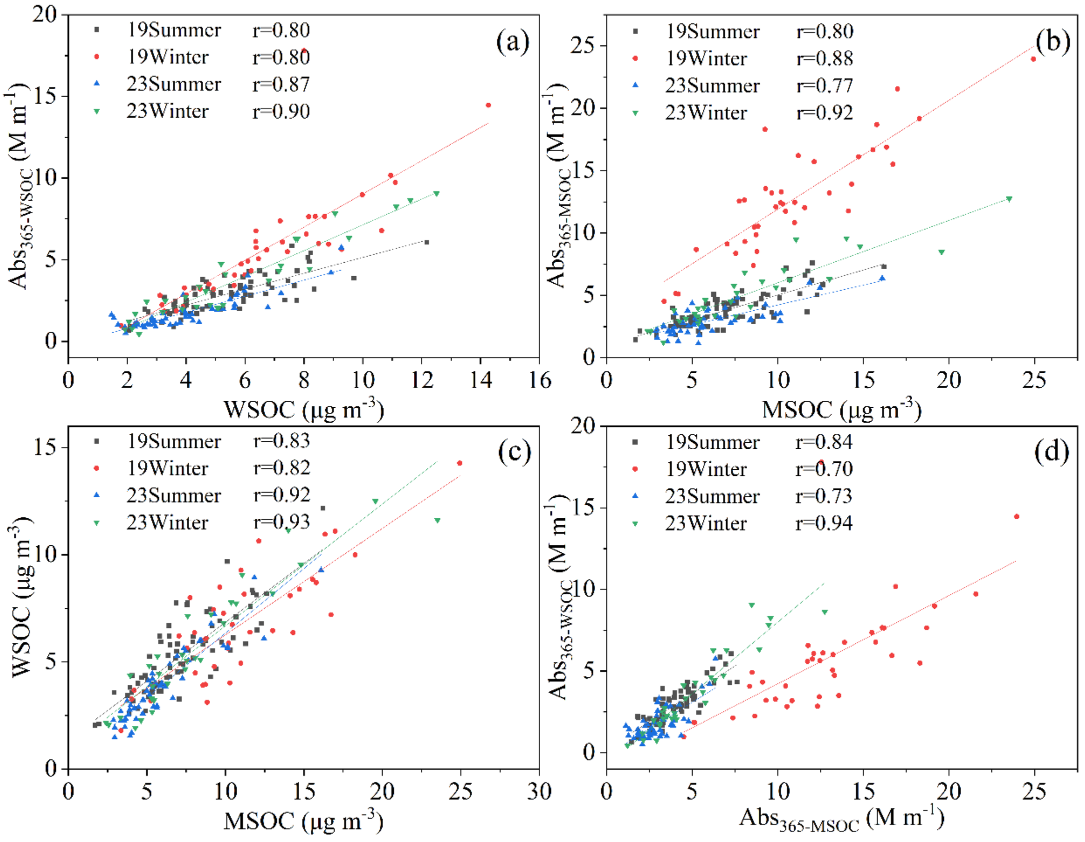

Figure S3. The relative signal fractions of the resolved fluorescent components derived from parallel factor analysis (PARAFAC) for (a) water-soluble organic carbon (WSOC) and (b) methanol-soluble organic carbon (MSOC) during different sampling periods.

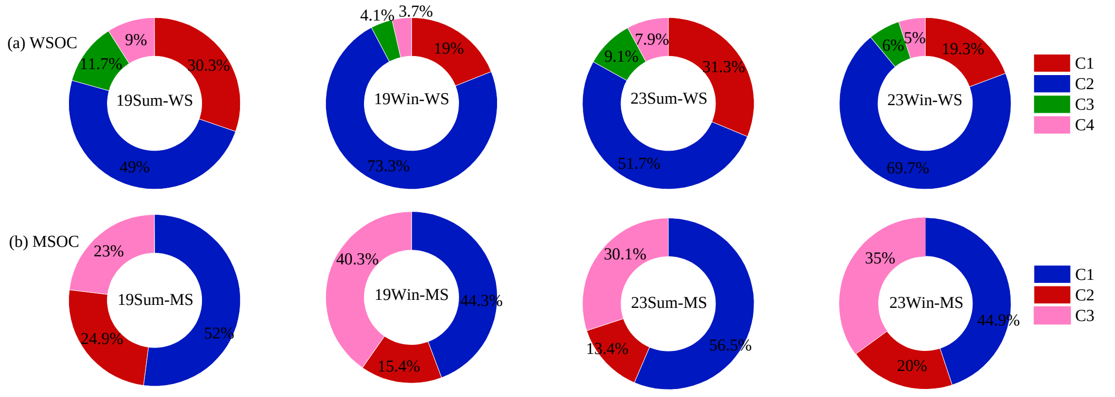

Figure S4. The relative signal fractions of different types of compounds in OA from different periods (meanings of 19SH, 19SL, 19WH, 19WL, 23SH, 23SL, 23WH, and 23WL are the same as those in Table S1).

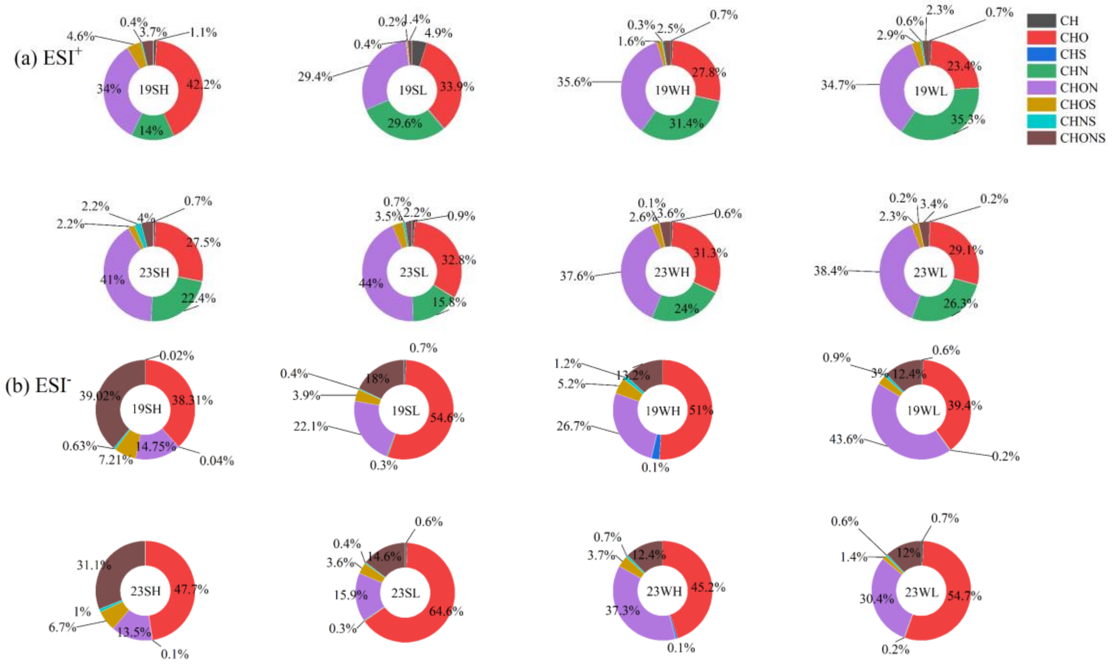

Figure S5. The Van Krevelen (VK) diagram of CHO compounds detected under ESI$^+$ mode. Different colors represent molecules with different aromaticity equivalent (Xc) values, and the pie chart shows corresponding number fractions (meanings of 19SH, 19SL, 19WH, 19WL, 23SH, 23SL, 23WH, and 23WL are as defined in Table S1).

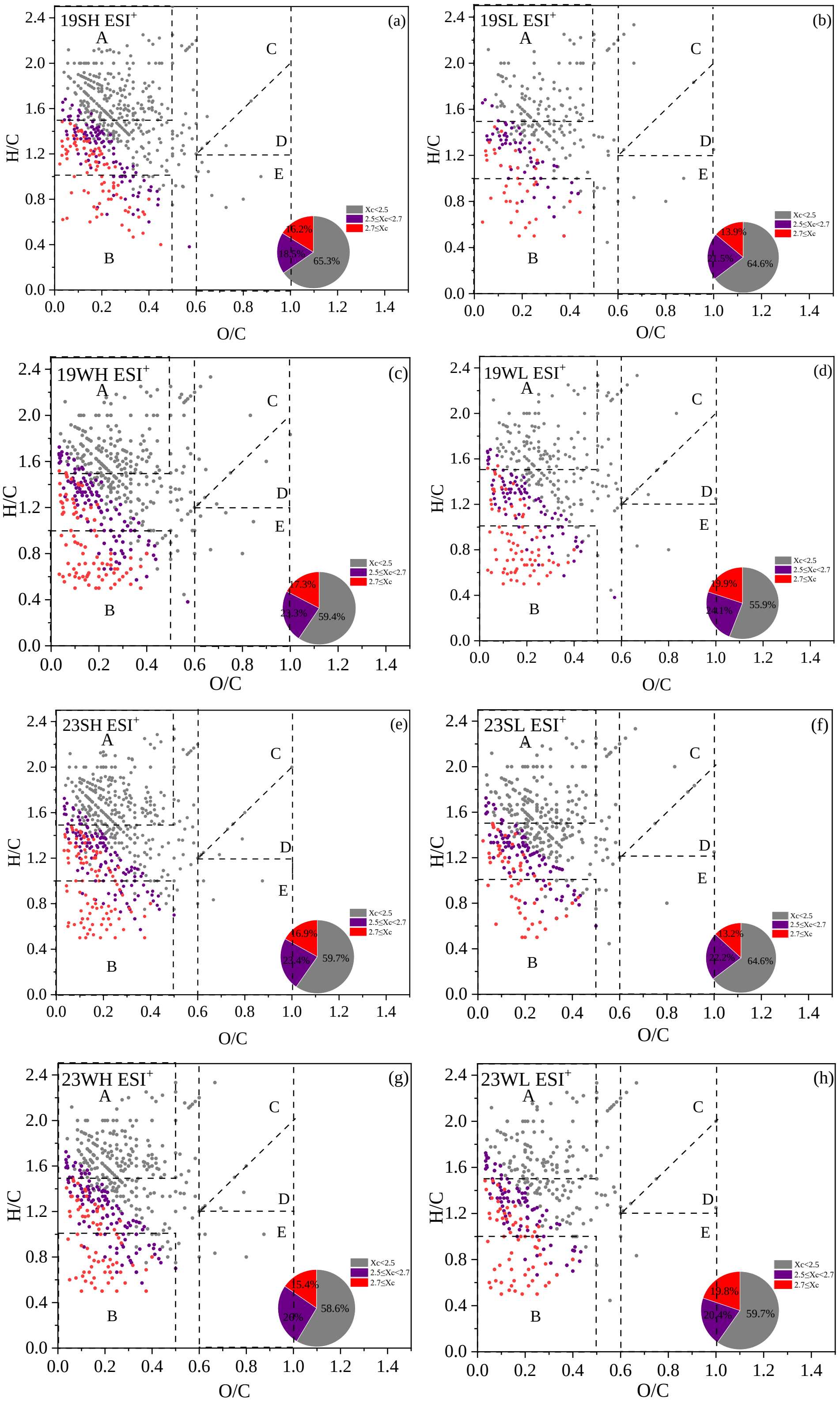

Figure S6. The Van Krevelen (VK) diagram of CHO compounds detected under ESI$^-$ mode. Different colors represent molecules with different aromaticity equivalent (Xc) values, and the pie chart shows corresponding number fractions (meanings of 19SH, 19SL, 19WH, 19WL, 23SH, 23SL, 23WH, and 23WL are as defined in Table S1).

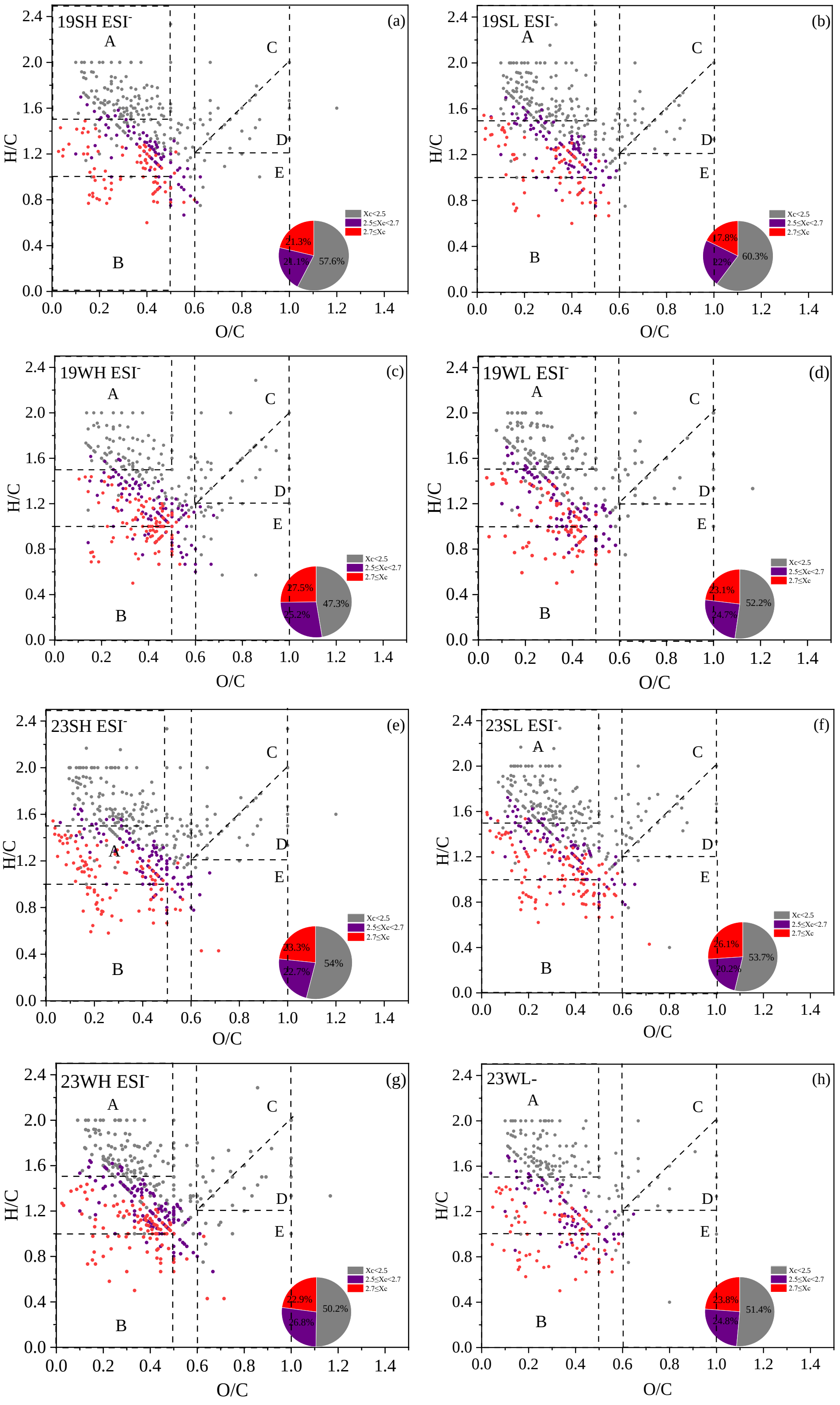

Figure S7. Carbon oxidation state (OSc) diagram of CHO compounds under ESI+ mode. The black dashed elliptical regions are labeled as hydrocarbon-like organic aerosols (HOA), biomass burning organic aerosols (BBOA), semi-volatile oxygenated organic aerosols (SV-OOA), and low-volatile oxygenated organic aerosols (LV-OOA) (meanings of 19SH, 19SL, 19WH, 19WL, 23SH, 23SL, 23WH, and 23WL are the same as those in Table S1).

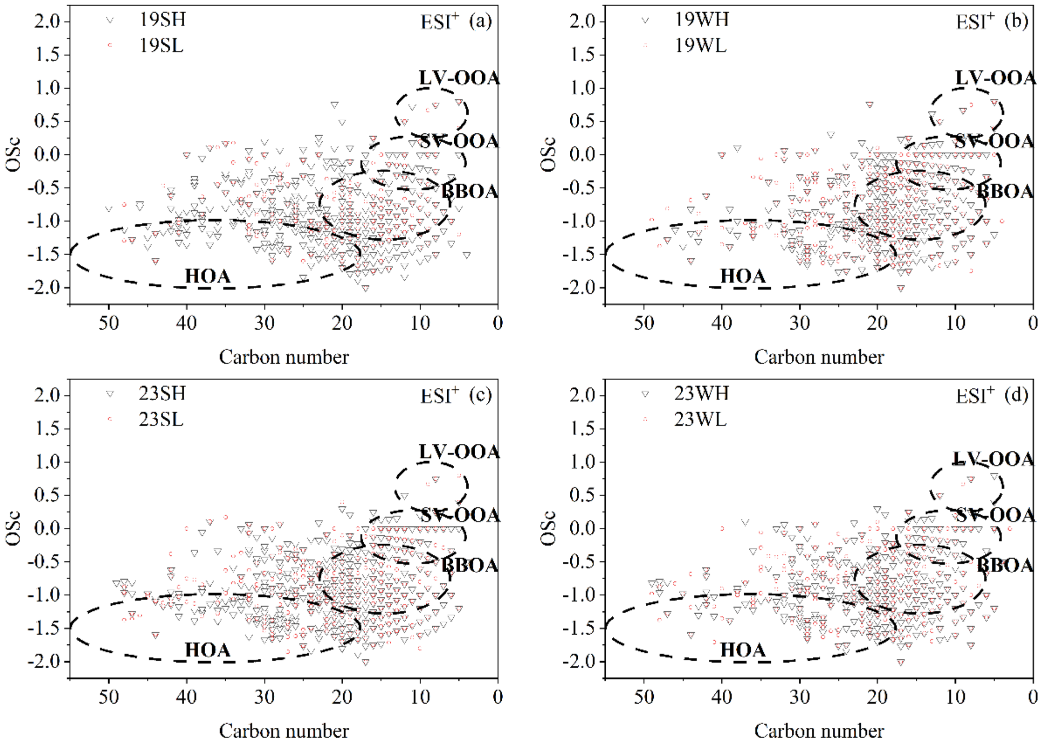

Supplement: Supplementary file 1 [file toxics-13-00443-s001.zip › toxics-3579782-supplementary.pdf]
